# A situation ignored in the healthcare: a systematic review of sexual harassment against healthcare professionals

**DOI:** 10.1590/1980-220X-REEUSP-2025-0419en

**Published:** 2026-10-12

**Authors:** Sinem Saricoban, Handan Alan, Hatice Ulusoy

**Affiliations:** 1 Sivas Cumhuriyet University, Faculty of Health Sciences, Department of Health Management, Sivas, Turquia.; 2 Istanbul University-Cerrahpaşa, Florence Nightingale Nursing Faculty, Department of Nursing Management, Istanbul, Turquia.

**Keywords:** Sexual Harassment, Health Personnel, Workplace Violence, Nurses, Assédio Sexual, Pessoal de Saúde, Violência no Trabalho, Enfermeiras e Enfermeiros

## Abstract

**Objective:**

The aim of this study was to systematically examine the findings of previous studies conducted in Türkiye on sexual harassment against healthcare professionals and to explore their experiences of sexual harassment.

**Method:**

The study, which was conducted with the systematic review method, was conducted within the framework of the PRISMA protocol, various keywords were searched in national and international databases and 16 studies were included in the review.

**Results:**

The exposure rate of sexual harassment varies between 0.0% and 75.0%. Female healthcare professionals, nurses, those aged 28–34 and those working in inpatient clinics are more likely to be subjected to sexual harassment. Healthcare professionals most frequently reported doctors as harassers. It has been found that healthcare professionals subjected to sexual harassment often did nothing and ignored the behavior.

**Conclusion:**

Further and in-depth research is needed to understand the experiences and coping methods of health workers who experience sexual harassment in Türkiye, and the impact of culture on them. Gender norms, stigmatization and workplace hierarchies often prevent reporting. Strategies should be developed by health policy makers to address these barriers.

## RESUMO

**Objetivo:**

O objetivo deste estudo foi examinar sistematicamente os achados de pesquisas anteriores conduzidas na Turquia sobre o assédio sexual contra profissionais de saúde e explorar suas experiências de assédio sexual.

**Método:**

O estudo foi conduzido por meio de uma revisão sistemática, seguindo as diretrizes do protocolo PRISMA. Foram realizadas buscas em bases de dados nacionais e internacionais utilizando diferentes palavras-chave, e 16 estudos foram incluídos na revisão.

**Resultados:**

A taxa de exposição ao assédio sexual varia entre 0,0% e 75,0%. Profissionais de saúde do sexo feminino, enfermeiros, aqueles com idade entre 28 e 34 anos e os que atuam em enfermarias de internação estão mais sujeitos ao assédio sexual. Os profissionais de saúde apontaram, com maior frequência, os médicos como autores do assédio. Verificou-se que os profissionais de saúde submetidos ao assédio sexual, frequentemente, não tomaram nenhuma atitude e ignoraram o comportamento.

**Conclusão:**

São necessárias pesquisas adicionais e mais aprofundadas para compreender as experiências e as estratégias de enfrentamento dos profissionais de saúde que vivenciam o assédio sexual na Turquia, bem como o impacto da cultura sobre eles. As normas de género, a estigmatização e as hierarquias no local de trabalho impedem frequentemente a denúncia. Os formuladores de políticas de saúde devem desenvolver estratégias para superar estas barreiras.

## INTRODUCTION

Violence is a significant public health issue that affects the lives of billions of people each year through death, injury, or illness^(1)^. With the global increase in violent incidents, it impacts individuals across various social strata and can arise in diverse settings, including households, public spaces, educational institutions, workplaces, and institutions. Workplace violence has become a worrying trend among workers from different professions and work environments worldwide^(2)^. Workplace violence can be defined as “Any act in which a person is abused, threatened, intimidated or assaulted in his or her employment”^(3)^. Occupational Safety and Health Administration (OSHA) defines workplace violence as “Workplace violence is any act or threat of violence against workers that can occur at or outside the workplace and ranges from threats and verbal abuse to physical assaults and homicide”^(4)^.

Workplace violence is a phenomenon that affects nearly all sectors and categories of workers; however, certain occupational groups are disproportionately exposed to a higher risk of such violence^(2,5)^. In particular, violence occurring in healthcare settings constitutes a significant public health concern and represents an increasingly important occupational health issue for healthcare workers^(2,3)^.

In the past ten years, there has been a notable increase in violence against healthcare professionals globally^(6)^. A study investigating violence against healthcare professionals, utilizing an umbrella review and meta-analysis approach, revealed that the overall rate of violence among healthcare professionals stood at 58.7%, comprising physical violence at 20.8%, verbal violence at 66.8%, and sexual harassment at 10.5%^(7)^. While sexual harassment is less common compared to other forms of violence, it remains a prevalent issue among healthcare professionals and has negative effects. Sexual harassment is a form of harassment that may include requests for sexual favors, offensive comments, or physical sexual behaviors^(8)^. An umbrella review of systematic reviews and meta-analyses to assess workplace violence against healthcare workers reported sexual harassment rates between 6% and 36.2%^(9)^. Similarly, another systematic review examining sexual harassment in female nurses found rates ranging from 10% to 87.3%^(10)^. Sexual harassment is a multifaceted phenomenon that encompasses unwanted verbal, visual, nonverbal, or physical behaviors of a sexual nature or based on an individual’s sex. Physical sexual harassment includes non-consensual behaviors such as unwanted touching, fondling, hugging, and kissing.

In contrast, verbal sexual harassment may take the form of sexually suggestive remarks, offensive comments, or sexually explicit jokes. Nonverbal sexual harassment encompasses behaviors such as intimidating or persistent staring, inappropriate gestures and facial expressions, as well as the display or transmission of sexually explicit photographs, images, or gifts. In addition, with the widespread use of information and communication technologies, cyber sexual harassment—including the sending of unwanted, offensive, and sexually explicit emails or text messages—has emerged as an increasingly prevalent form of sexual harassment^(11)^.

Studies investigating sexual harassment among healthcare professionals indicate that individuals subjected to sexual harassment often report physical, psychological, and negative work-related symptoms following such incidents^(12,13,,14)^.

Systematic and meta-analysis studies examining sexual harassment against healthcare professionals can be found in the international literature^(10,15)^. However, no systematic study has been encountered on this issue in Türkiye. While patriarchal norms and traditional gender roles increase the risk of sexual harassment, nurses and other female healthcare workers are particularly vulnerable to harassment perpetrated by patients, patients’ relatives, and even colleagues. Furthermore, sociocultural and legal barriers, including stigmatization, victim-blaming attitudes, and the burdensome nature of legal procedures, may discourage victims from reporting such incidents to official authorities^(16,17)^. Structural and organizational factors within the healthcare system, including high patient loads, long working hours, and inadequate security measures (especially during night shifts), deepen existing structural risks by rendering staff more vulnerable to patients and their relatives^(18)^. Considering all these reasons, this study investigates the role of Türkiye-specific cultural and contextual determinants in shaping the prevalence, risk, and protective factors of sexual harassment among healthcare workers by focusing on the incidents of sexual harassment experienced by healthcare workers in Türkiye, rather than the extensive sexual harassment studies in the global literature. The findings are expected to enhance understanding of local realities, contribute to cross-cultural interpretations of sexual harassment, and inform the development of context-specific health policies and prevention strategies. In this systematic review, the aim was to systematically examine the findings of previous studies conducted in Türkiye related to sexual harassment against healthcare professionals. It was also aimed to find out healthcare professionals’ sexual harassment experiences.

## METHOD

### Study Type, Protocol and Registration

This study was conducted using the systematic review method and followed Joanna Briggs Institute (JBI) protocol^(19)^. Preferred Reporting Items for Systematic Reviews and Meta-Analyses (PRISMA) was followed in the creation of the study protocol and writing of the report^(20)^. The study protocol was registered to PROSPERO (Registration number: CRD42023421654).

### Review Questions

This systematic review sought to elucidate the following research questions:

Regarding healthcare professionals;

- What are the findings regarding the sexual harassment they were exposed to?
- What findings have been reported following sexual harassment?
- What are the recommendations for the prevention of sexual harassment?

The compilation question is structured according to the PCC (Population–Concept–Context) framework. Accordingly, the **population** includes all healthcare workers such as physicians, nurses, midwives, and healthcare technicians working in Türkiye; the **concept** includes all types of sexual harassment, including verbal, physical, psychological harassment, and mobbing; and the **context** covers all healthcare institutions in Türkiye.

### Eligibility Criteria

This systematic review examined research articles reporting findings related to sexual harassment among healthcare workers in Türkiye. The study included all forms of violence, irrespective of type; studies addressing sexual harassment alongside other types of violence were also included. Original research articles published in Turkish or English, with full text available, have been included in the scope of the study. No restrictions were applied in terms of publication year, type of sexual harassment, occupational group, or type of healthcare institution. Within the scope of exclusion criteria, publications whose full text is unavailable, studies published in languages other than Turkish or English, conference papers, notes, conference reviews, book chapters, editorials, brief surveys, letters, and review articles have been excluded from evaluation.

### Search Strategy

Within the scope of the study, the research published until 31 December 2023 was indexed using five national and international databases in English keywords in accordance with the “Medical Subject Headings (MESH)” and Turkish keywords in accordance with the Turkish Science Terms on PubMed, Science Direct, Emerald, Web of Science, and DergiPark.” (Table 1). Keywords were searched by combining them using “AND” in each database. In the Türkiye-based database DergiPark: The keyword combinations “Cinsel Taciz AND Doktor AND Türkiye”, “Cinsel Taciz AND Hekim AND Türkiye”, “Cinsel Taciz AND Hemşire AND Türkiye”, “Cinsel Taciz AND Sağlık Personeli AND Türkiye”, “Cinsel Taciz AND Sağlık Çalışanı AND Türkiye” were used. Once again, searches were conducted in English using the Türkiye-based database DergiPark and other databases such as PubMed, Science Direct, Emerald, and Web of Science, using the search terms “Sexual Harassment AND Doctors AND Turkey”, “Sexual Harassment AND Physicians AND Turkey”, “Sexual Harassment AND Nurses AND Turkey”, “Sexual Harassment AND Health Staff AND Turkey”, “Sexual Harassment AND Healthcare Staff AND Turkey”, “Sexual Harassment AND Health Care Staff AND Turkey”, “Sexual Harassment AND Health Professionals AND Turkey”, “Sexual Harassment AND Healthcare Professionals AND Turkey”, “Sexual Harassment AND Health Care Professionals AND Turkey”, “Sexual Harassment AND Health Workforce AND Turkey”, “Sexual Harassment AND Healthcare Workforce AND Turkey”, “Sexual Harassment AND Health Care Workforce AND Turkey”, “Sexual Harassment AND Health Personnel AND Turkey”, “Sexual Harassment AND Healthcare Personnel AND Turkey”, “Sexual Harassment AND Health Care Personnel AND Turkey”, “Sexual Harassment AND Health Workers AND Turkey”, “Sexual Harassment AND Healthcare Workers AND Turkey”, “Sexual Harassment AND Health Care Workers AND Turkey”.

**Table 1:** Databases and keywords – Türkiye, 2024.

Databases	Turkish keywords	English keywords
DergiPark	Cinsel taciz, doktor, hekim, hemşire, sağlık personeli, sağlık çalışanı, Türkiye	
PubMed, Science Direct, Emerald, Web of Science		Sexual harassment, doctors, physicians, nurses, health staff, healthcare staff, health care staff, health professionals, healthcare professionals, health care professionals, health workforce, healthcare workforce, health care workforce, health personnel, healthcare personnel, health care personnel, health workers, healthcare workers, health care workers, Turkey

### Selection Process

The EndNote X9 program, used to manage bibliographic information, was used to collect studies from the databases. In EndNote, a library was created for each database, then the libraries were combined, and duplicate studies were excluded. The screening process was carried out simultaneously and independently by two authors (SS, HA), the titles and abstracts of the studies were examined, and the authors compared all the resulting studies. At the next stage, all authors evaluated full-text studies according to the inclusion and exclusion criteria. At the last stage, 16 articles were included in the systematic review. The process was described in detail in the PRISMA flow chart (Figure 1).

### Methodological Quality Assessment of the Studies

The quality assessment of the selected studies was performed using the JBI Analytical Cross-Sectional Checklist. The checklist consists of 8 questions, and each question is evaluated as “yes,” “no,” “unclear,” or “not applicable.” Two researchers (SS, HA) independently conducted evaluations, with a third researcher (HU) resolving discrepancies. In the quality assessment, the number of items rated as ‘yes’ out of eight was considered the quality score (QS).

### Literature Review

To reduce the risk of possible bias during the study, the literature review, selection of articles, data collection, and evaluation of article quality procedures were performed by two researchers (SS and HA) independently, and each stage of the process was checked by the third researcher (HU), and consensus was reached. In addition, three researchers (SS, HA, HU) participated in a pilot study to perform these operations correctly and successfully. This pilot study involved reviewing the articles, collecting data, and evaluating their quality. The differences of opinion that emerged after the pilot study were resolved through discussion.

### Data Collection

A data collection tool developed by the researchers was used to obtain the data in the study. With this data collection tool, data related to the author(s) and the year of publication, the place where the study was conducted, the characteristics of the participants and the status of sexual harassment were obtained.

### Ethical Approval

Ethical approval was not required because the data were secondary data obtained from the databases. This study did not involve experiments on humans or animals, and no individual-level participant data were collected.

### Data Analysis

The methodology, the characteristics of the participants, the evaluation methods and the outcome variables of the studies included in the systematic review vary accordingly. Therefore, the findings were tabulated and presented in narrative form since meta-analysis could not be performed.

### Limitations

The fact that the vast majority of the studies examined have a cross-sectional study design and that data are collected through self-reporting limits causal inference and increases the risk of response bias. The lack of a standard classification of sexual harassment types and occupational groups has led to significant methodological heterogeneity across studies, making it difficult to compare prevalence estimates directly. Furthermore, the failure to use validated and reliable measurement tools in every study and the preference for non-standardized questionnaire forms developed by researchers in some studies has increased the risk of measurement error and systematic bias. The self-report-based data collection process is susceptible to recall bias, depending on how participants remember their past experiences, and may affect the accuracy of reported prevalence rates. However, the fact that a significant portion of the studies were conducted in Ankara (the capital of Türkiye) indicates regional concentration; the absence of nationally representative samples limits the generalizability of the findings to Türkiye as a whole.

Furthermore, given that sexual harassment is a stigmatizing phenomenon, participants may be inclined to underreport or conceal their experiences. Therefore, the reported prevalence rates may underrepresent the actual situation. Finally, the fact that the studies were predominantly conducted in secondary and tertiary healthcare institutions, coupled with the lack of research covering primary healthcare services and the private healthcare sector, is another important factor limiting the generalizability of the findings.

## RESULTS

### Screening Findings

Initial database searches identified 5,169 articles. After removing duplicates in EndNote X9, 890 records remained. Title/abstract screening narrowed these to 91 articles for full-text review. Of these, 16 met inclusion criteria by reporting healthcare professionals’ sexual harassment experiences (Figure 1). These 16 studies were included in the final analysis.

**Figure 1:**
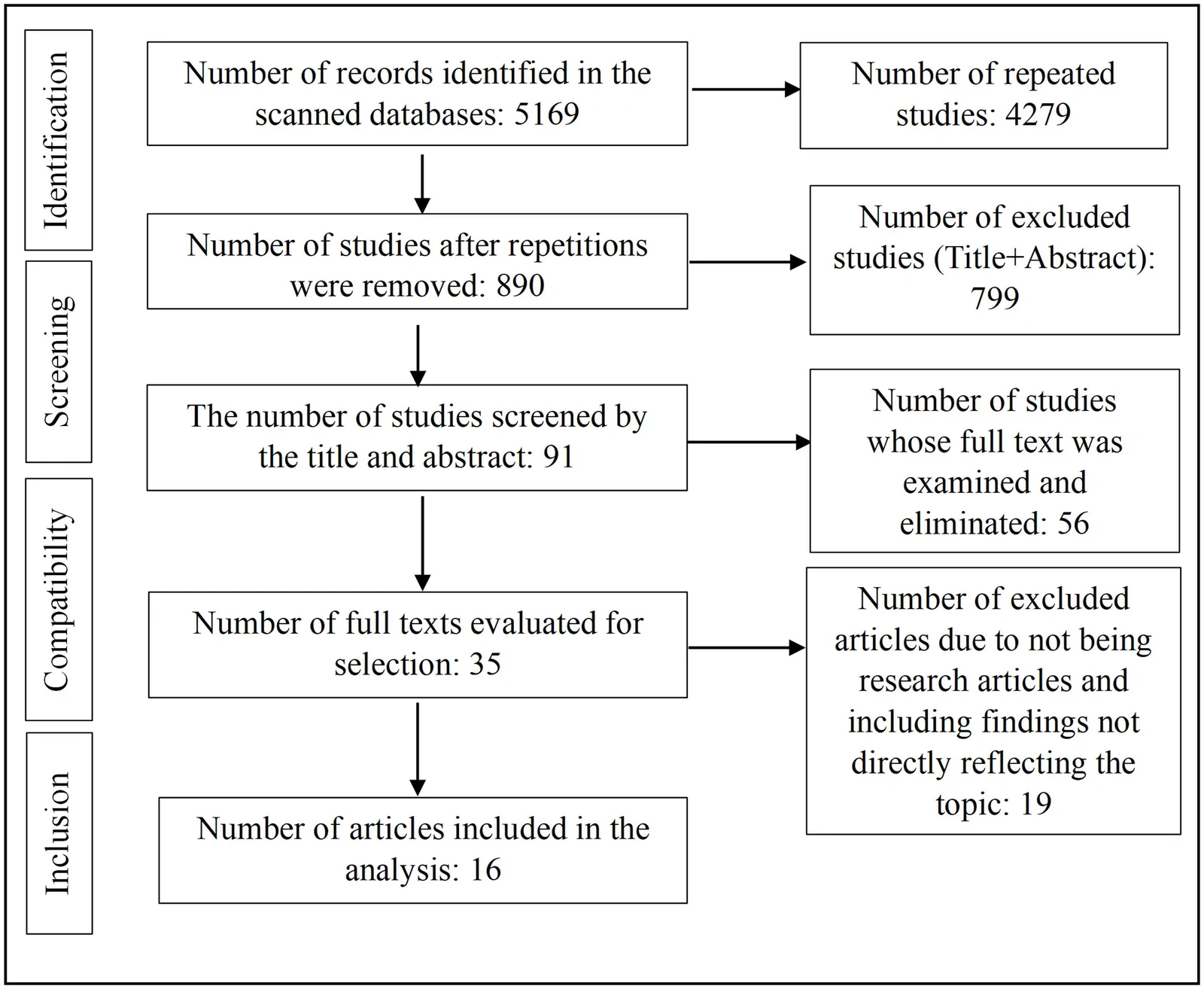
PRISMA flow diagram – Türkiye, 2024.

### General Characteristics of the Studies

The studies included in the review were conducted between 1996 and 2023; 15 were descriptive quantitative studies, while descriptive quantitative and qualitative methods were used in one study^(18)^. While two studies^(21,22)^ were conducted throughout Türkiye, five were conducted in Ankara^(23,24,25,26,27)^, and the city/region was not specified in 5 studies^(18,28,29,30,31)^. Each of the remaining studies was carried out in different cities^(32,33,34,35)^.

When the institutions where the studies were conducted were examined, eight studies were conducted in public hospitals^(23,24,27,30,31,33-35)^, eight studies at training and research hospitals^(25,27,29-33,35)^, and two studies at private hospitals^(31-33)^. The institutions where the studies were carried out were not specified in four articles^(18,22,26,28)^. In one study, it was stated that the research was conducted in primary, secondary, and tertiary health institutions^(21)^.

A total of 11 studies were conducted throughout the hospital^(18,21,23-26,28-30,34,35)^, four in emergency departments^(22,27,32,33)^, and one in the operating room^(31)^.

Eight of the studies were conducted with nurses^(23,25,26,29-31,34,35)^, six were conducted with all healthcare professionals^(21,24,27,28,32,33)^, and two were conducted with physicians^(18,22)^. The total sample size of the studies included 18,014 healthcare professionals, and the sample size of each study ranged from 49 to 12,944.

In six of the studies, only sexual harassment in healthcare professionals was investigated^(18,23,25,26,30,35)^, while it was considered together with other types of violence in 10 studies^(21,22,24,27-29,31-34)^ (Table 2).

**Table 2:** Bibliographic characteristics of the research (N = 16) – Türkiye, 2024.

Variable		n
Type of study	Quantitative	15
	Quantitative+ Qualitative	1
The place (city) where the study was conducted*	Ankara	5
	Throughout Türkiye	2
	Unspecified	5
	Other	4
The institution where the study was conducted*	Training and Research Hospital	8
	Public Hospital	8
	Private Hospital	2
	Unspecified	4
The unit where the study was conducted	Hospital	11
	Emergency Service	4
	Operating Room	1
Sample Group	Nurse	8
	All healthcare professionals	6
	Physician	2
Number of samples	≤200	6
	≥201	10
The scope of the research	Sexual harassment/sexual violence	6
	All types of violence	10

*Some of the studies were carried out in more than one city and institution.

### Data Collection Tools Used in the Studies

Questionnaires were used as a data collection tool in all of the studies. Different scales developed in other studies were used in three studies^(24,26,29)^. Forms created based on questionnaire forms published by organizations such as the World Health Organization (WHO), the International Labor Organization (ILO), the International Nurses Association (ICN), and the American Medical Association (AMA) were used in three studies^(21,28,34)^, while the questionnaire form created by the researchers using similar studies in the literature was used in 10 studies^(18,22,23,25,27,30-33,35)^ (Table 3).

**Table 3:** Characteristics of the studies addressing sexual harassment against healthcare professionals in Türkiye – Türkiye, 2024.

Study no.	Author(s), year and QS	Place of the study	Research design	Sample	Data collection tools	Key findings
1	Kisa and Dziegielewski 1996^(25)^, QS:6/8	1 training hospital	Descriptive	184 nurses	Cholewinski & Burge’s (1990) questionnaire form created based on the Sexual Harassment Inventory and the Phillips & Schneider (1993) questionnaire, (18 multiple choice questions)	75% of nurses reported being subjected to sexual harassment. The most common types were sexual jokes (72%), obscene gestures (65%), and coercion for a date (53%). Harassers were mostly doctors (43%) and patients (34%). 39% experienced harassment 1–4 times during their career, with 41% occurring during night shifts (16:00–12:00). Common responses included ignoring the behavior (29%), telling the person to stop (26%), joking (18%), and avoidance (18%). Harassment mostly occurred in patient rooms (32%), non-clinical areas (30%), and hospital wards (20%). 47% reported reduced productivity. Emotional responses included anger (44%), humiliation (14%), and fear (12%), while physical symptoms were headaches (38%), insomnia (20%), and fatigue (15%).
2	Kisa et al. 2002^(30)^, QS:6/8	1 university and 1 public hospital	Descriptive	215 nurses	The questionnaire form created by Kısa and Dziegielewski (1996) (18 multiple choice questions)	73% of nurses reported exposure to sexual harassment. Common types included insinuating glances (20%), sexual jokes (19%), and obscene gestures (18%). Harassers were mostly doctors (41%) and patients (39%). 38.4% of incidents occurred during night shifts. Harassment mainly took place in patient rooms (34%), non-clinical areas (30%), and hospital services (18%). Nurses often responded by ignoring the behavior (29%), avoiding the harasser (21%), or joking about it (15%). 45% reported decreased productivity. Emotional responses included anger (42.9%), fear (14.1%), and humiliation (10.5%). Physical symptoms were headache (37.7%), insomnia (20.2%), and gastritis (12.9%).
3	Ayrancı 2005^(33)^, QS:6/8	Emergency departments	Descriptive	195 health personnel	The questionnaire form created by the researchers based on the literature	The emergency service personnel who participated in the study stated that they had never been subjected to sexual harassment before.
4	Çelik and Çelik, 2007^(23)^, QS:8/8	8 public hospitals	Descriptive	622 nurses	A questionnaire created by the researchers based on the literature and the American Medical Association	The prevalence of sexual harassment among nurses is 37.1%. Higher rates were reported among single nurses (42.3%), nursing graduates (52.8%), those aged 30–34 (45.7%), with 10–14 years of experience (47.7%), and those working in inpatient units (39.6%). Harassers were mainly physicians (77.1%), other nurses (51.1%), and patients (43.3%). The most common form was unwanted sexual jokes, stories, or comments. 45% reported reduced productivity; 37.2% considered quitting; 36.8% noted harm to social life, 27.3% to family life; 44.6% experienced mental health issues, 40.3% headaches, and 24.2% physical health problems. Common reactions included doing nothing (59.3%), setting boundaries (43.3%), and ignoring the incident (30.7%).
5	Erdemir et al. 2011^(35)^, QS:7/8	1 university hospital, 2 public hospitals	Descriptive (quantitative and qualitative)	184 nurses	The questionnaire form created by the researchers based on the literature (13 questions)	71.7% of nurses reported being sexually harassed by patients. The most common forms were uncomfortable staring (61.4%), intrusive personal questions (34.8%), and sexual threats or profanity (26.1%). After the incident, 31.4% acted as if nothing happened, 29.3% sought help from colleagues or managers, and 18.5% directly rejected the behavior. Nurses’ suggestions for prevention included self-restriction, limiting communication with patients, enhancing security and supervision, providing education and awareness, and modifying or terminating patient care.
6	Talas et al. 2011^(27)^, QS:6/8	Emergency departments	Descriptive (quantitative and qualitative)	270 emergency service personnel	The questionnaire form created by the researchers based on the literature (The 36-item questionnaire -28 close-ended and 8 open-ended-)	15.9% of healthcare professionals reported experiencing sexual harassment. Higher rates were found among women, nurses, college graduates, and those aged 28–34. Common forms included unwanted sexual jokes or comments (51.2%), suggestive gestures (37.2%), and unwanted date offers (39.5%). Harassers were mostly patients’ relatives, patients, doctors, and nurses. Reactions included doing nothing (37.2%), leaving the scene (37.2%), and reporting the incident (34.9%). The most common emotional responses were sadness, anger, and frustration.
7	Ulusoy et al. 2011^(18)^, QS:6/8	A 1000-bed hospital	Descriptive (quantitative and qualitative)	49 assistant physicians	The questionnaire created by the researchers (12 Yes ⁄No items)	67.3% of female assistant physicians reported experiencing sexual harassment. Common forms included uncomfortable staring (51.0%), sexually explicit questions and date offers (both 14.3%). Coping strategies included requesting patient discharge (24.2%), cutting ties with the harasser (24.2%), rejecting the behavior (21.2%), ignoring the incident (21.2%), and seeking help (12.1%). Prevention measures focused on patient-physician interaction, appearance, avoiding being alone with patients, and using physical barriers between physicians and patients or their relatives.
8	Tekin and Bulut 2014^(31)^, QS:8/8	17 Public, 8 university, 13 private hospitals	Descriptive	380 surgical nurses	The questionnaire form created by the researchers based on the literature (48 questions)	32.6% of operating room nurses reported experiencing sexual harassment, mostly by doctors (94.4%), colleagues (41.1%), and other staff (20.2%). The most common forms were unwanted jokes/comments (89.5%), suggestive gestures (52.4%), and pressure to maintain relationships outside of work (26.6%). Emotional responses included anger (50%), surprise (28.2%), and humiliation (25%). While 40.3% downplayed the incident, others felt they could cope (22.6%), blamed themselves (11.3%), or believed they didn’t deserve it (5.6%). Post-incident reactions included ignoring it (40.3%), confronting the harasser (21%), discussing it (16.1%), cutting off communication (10.5%), and reporting it to managers.
9	Pınar et al. 2017^(21)^ QS:8/8	Primary, secondary and tertiary healthcare professionals	Descriptive	12,944 medical personnel	Country Case Studies of Workplace Violence in the Health Sector Research Tools-Questionnaire created by the ILO, ICN, WHO, PSI (130 items)	1% (n = 130) of healthcare professionals reported exposure to sexual harassment, including 11 from primary care and 119 from secondary care. Of these, 28 were men and 102 women; 71 were nurses/midwives, 19 physicians/dentists, 16 EMS staff, 16 technicians, and the rest from various roles. 110 worked in the public sector and 19 in the private sector. Harassment was mostly by patients (38.2%), patient relatives (36%), and coworkers (30.8%). Perpetrators were predominantly male (78.5%).
10	Aydemir et al. 2020^(32)^, QS:7/8	Medical school emergency department	Descriptive	121 healthcare professionals	The questionnaire form created by the researchers based on the literature (34 questions)	The rate of sexual harassment of emergency service personnel is 1.8%.
11	Demirci and Uğurluoğlu 2020^(24)^, QS:8/8	1 public hospital	Descriptive	347 healthcare professionals	The questionnaire form created by Çelik and şenol-Çelik (2007) (42 items)	The overall rate of sexual harassment among healthcare professionals is 16.4%, with higher rates among nurses (26.9%) compared to physicians (16.3%) and other personnel (10.0%). Physicians were the main harassers for 12.5% of physicians, 22.6% of nurses, and 8.0% of other staff. Harassment led to decreased productivity (35.1%) and thoughts of quitting (21.1%). It also impacted social (22.8%) and family life (14.0%). Physical effects included headaches (29.8%), health deterioration (22.8%), and abdominal pain (12.3%); psychological effects included anger (64.9%), mental health decline (42.1%), and fear (28.1%). Common coping methods were taking no action (82.5%), confronting the harasser (52.6%), and ignoring the behavior (49.1%). Harassment negatively impacted nurses’ quality of work life and increased their intention to leave.
12	Kırlıoğlu ve Toygar, 2020^(26)^ QS:8/8	In various hospitals	Descriptive	571 nurses	The scale developed by Turgut (2007) (22 items)	It has been found that the exposure of nurses to sexual harassment adversely affects their quality of work life and their intention to leave work.
13	Çam and Üstüner-Top 2021^(34)^, QS:8/8	1 public hospital	Descriptive	865 nurses	WHO, ILO, ICN and PSI created the “Workplace Violence in the Health Sector Questionnaire”	Sexual harassment accounted for 1.7% of all violence experienced by nurses. Ten female and five male nurses reported being harassed, mostly by hospital staff (26.7%), patient relatives (20%), and colleagues (20%). Most incidents occurred within the hospital (60%). Common reactions included telling the harasser to stop (33.3%), informing friends/family (33.3%), and pretending it didn’t happen (20%). 73.3% believed the incident was unavoidable. Reasons for not reporting included fear of consequences (40%), shame (33.3%), and believing it wouldn’t help (26.7%).
14	Dursun and Aytaç 2021^(28)^, QS:8/8	n/a	Descriptive	169 healthcare professionals	Workplace Violence Questionnaire developed by WHO and ILO and adapted by Aytaç et al. (2011)	5.4% of healthcare professionals reported sexual harassment, all of whom were women. No male healthcare professionals experienced harassment. Harassers were mostly men (87.5%). Those exposed to violence reported lower job satisfaction compared to those who were not.
15	Sabak et al. 2021^(22)^, QS:6/8	All emergency physicians affiliated with an association	Descriptive	362 emergency physicians	The questionnaire form created by the researchers based on the literature (34 Questions)	The sexual harassment rate among emergency room physicians is 6.1%. Among those affected, 6.7% were men and 5.1% women. Harassers included patients (3.3%), family members (3.3%), and intoxicated individuals (2.2%). 96.6% reported a lack of hospital rules restricting patient and visitor movement. Additionally, 59.1% noted the absence of security personnel, 100% said guards were unarmed, and 95.5% felt inadequately protected.
16	Erkutlu et al. 2023^(29)^, QS:8/8	5 private, 5 public affiliated university hospitals	Descriptive	536 nurses	The scale developed by Ding et al., 2016 (7 items, six-point Likert scale)	The rate of sexual harassment in nurses is 0.4%.

QS: Quality Score.

### Findings on the Status of Sexual Harassment of Healthcare Professionals

#### Prevalence and Types of Sexual Harassment

In the studies examined in this systematic review, rates of sexual harassment among health workers ranged from 0.0% to 75.0%, and it was found to be higher than 70.0% in three studies where only sexual harassment was investigated^(25,30,35)^. In studies conducted together with other types of violence, participants reported that they were subjected to verbal abuse/violence the most (38.4%–99.0%), while they reported that they were subjected to sexual harassment the least (0.0%–32.6%)^(21,22,24,27-29,31-34)^.

When the samples were examined, while the exposure rate of sexual harassment was reported between 0.0–16.4% in the studies conducted with all healthcare professionals^(21,24,27,28,32,33)^, it was between 0.4% and 75.0% in studies conducted only with nurses^(23,25,29-31,34,35)^, 67.3% in studies conducted with female assistant physicians^(18)^ and 6.1% in studies conducted with emergency medicine physicians^(22)^. When some personal and professional characteristics of healthcare professionals who had been subjected to sexual harassment were examined; female healthcare professionals were subjected to sexual harassment more compared to male employees^(21,24,27,28,34)^, while it was nurses compared to other health professionals^(21,24,27)^. The studies were conducted with healthcare professionals between the ages of 18–51 and between the ages of 28–34^(23,27)^ and those who were single, had less professional experience, and worked in inpatient clinics had a higher rate of exposure to sexual harassment^(23)^.

The frequency of exposure to sexual harassment was reported in four of the examined studies^(23,25,28,30)^ and the proportion of healthcare professionals who reported being subjected to sexual harassment at least once during their careers^(25)^ ranged from 11.0% to 77.8%^(28)^.

Within the scope of the studies examined, the majority of the participants stated that they were subjected to verbal sexual harassment such as sexually suggestive words, jokes and stories, sexual threats or use of profanity, offers to meet outside after work, asking questions about private life and unsolicited phone calls or emails^(18,23,25,27,30,31,35)^. In addition, participants also mentioned physical sexual harassment behaviors such as sexually suggestive exposure of some parts of the body, rubbing, touching, hugging, holding, insinuating glances, attempted sexual assault, staring uncomfortably, trying to show their genitals, stalking outside work, asking to be touched on the genitals by a healthcare professional, and trying to pull them to bed^(18,23,25,27,30,31,35)^.

### Characteristics of Victims and Perpetrators

In the studies examined in the systematic review, participants reported that many people sexually harassed them in their work environments. In five of these studies, participants reported that they were most often harassed by physicians^(23-25,30,31)^. In contrast, patients were reported to be the perpetrators in two studies^(21,22)^, patient relatives in one study^(27)^, and hospital staff in one study^(34)^. A study conducted by Erdemir et al.^(35)^ investigated sexual harassment experienced by nurses only by patients, and a study conducted by Ulusoy et al.^(18)^ investigated sexual harassment experienced by female assistant physicians only by patients and patient relatives. In addition, intern doctors, faculty members, cleaning or maintenance personnel, security personnel, other colleagues, and managers were also identified as harassers^(21,23,25,30,31,34)^. Sabak et al.^(22)^ emphasized that emergency room physicians were also sexually harassed by drunks and mentally ill people in addition to the above-mentioned groups.

### Contextual Factors (Work Setting, Shift Patterns, Institutional Characteristics)

Participants in the studies reported that sexual harassment occurred most often in patient rooms, non-clinical settings or inpatient clinics^(25,30,34)^. They also stated that they were most exposed to sexual harassment during the night shift^(25,30)^.

### Responses and Reporting Behaviour

In the studies examined, healthcare professionals who were subjected to sexual harassment often did nothing^(23,24,27,35)^ and acted as if it had not happened and ignored the behavior^(25,30,31,35)^. Ulusoy et al.^(18)^ determined that female assistant physicians wanted the patient to be discharged in such a situation and cut off communication. A very low percentage of participants stated that they talked about the situation with a friend or manager when they were subjected to sexual harassment^(18,23-25,27,30,35)^. At the same time, only one study emphasized that healthcare professionals did not tell or report the incident to anyone else because they were afraid of negative consequences, ashamed, felt guilty, and thought it would not work^(34)^.

The studies considered within the scope of the review reported that individuals were affected physically and emotionally/psychologically following sexual harassment and that these effects led to negative work-related consequences. Participants physically experienced headaches, inability to sleep or sleeping more than usual^(18,23-25,27,30,35)^ and emotionally/psychologically experienced anger, humiliation, fear, guilt, self-blame, and feeling humiliated frequently^(23-25,27,30,31)^. As a result, healthcare professionals’ productivity and quality of work life decreased, and they considered quitting their jobs^(23,24,25,26,30,31)^.

### Recommendations of Healthcare Professionals for the Prevention of Sexual Harassment

In two of the studies examined, healthcare professionals who were subjected to sexual harassment made recommendations related to *(1)* patients and healthcare staff and *(2)* the institution to avoid sexual harassment experienced at work and for prevention^(18,35)^.

(1) *Recommendations related to patients and healthcare professionals* included terminating the treatment and discharging the patient, providing psychological support to the patient, not being alone with the patient in the room, limiting the time allotted to the patient, not making direct eye contact with the patient or relatives, being harsh with the patient and relatives, approaching the patient and relatives professionally, referring to the patient and relatives as ‘sir’ instead of ‘uncle’ or ‘brother’, keeping communication short, being serious and not making jokes, avoiding mistakable behavior, avoiding attention grabbing clothing, preferring modal and baggy clothes as possible and not wearing excessive makeup, keeping physical distance from patients and relatives as much as possible, and ensuring that their peers take care of patients with tendency to harassment (for instance, a male colleague taking care of a male patient)^(18,35)^.
(2) *Recommendations related to the institution included* hanging informative and stimulating posters about the rights of healthcare professionals, installing and disseminating a camera system, having a security guard particularly during the night shift, increasing the number of security personnel, ensuring that security personnel frequently visit polyclinics and patient rooms, organizing training for staff to be informed about this situation, ensuring that the staff is prepared for unexpected situations, carefully preparing the working hours of female employees and preventing them from working alone at night, increasing the number of medical personnel and preventing female personnel from being alone with patients and relatives, supporting the employee instead of the harasser as a manager, introducing strict rules for patient visits, ensuring that the abuser faces punishment^(18,35)^.

## DISCUSSION

This systematic review evaluated 16 articles (1996–2023) on sexual harassment experiences among healthcare professionals in Türkiye. Most studies were regionally focused (mostly Ankara), with few nationwide samples, limiting generalizability of prevalence data. While the majority examined the Ministry of Health hospitals, some omitted institutional details—likely to protect participant confidentiality. This aligns with broader literature noting that national-scale studies often concentrate on specific cities, hospitals, or specialties^(12)^.

Most of the questionnaire forms used in the studies include different measurement tools created by the authors. It can be concluded that there is no common measurement tool widely used in the evaluation of sexual harassment in healthcare professionals. Therefore, evaluation methods and outcome variables vary across studies.

In most of the studies analyzed, sexual harassment was investigated in addition to other forms of violence, and it was found that all studies reported high rates of sexual harassment as well as no sexual harassment. In the systematic review study conducted by Lu et al.^(36)^, which examined the frequency of sexual harassment against nurses worldwide, it was found that the rate of sexual harassment in nurses was between 12.6% and 53.4%. A study conducted among social and healthcare workers in Germany found that 62.5% of participants had experienced nonverbal sexual harassment, 67.1% had been subjected to verbal sexual harassment, and 48.9% had encountered physical sexual harassment perpetrated by patients, clients, or residents in the workplace^(37)^. In Italy, the prevalence of sexual harassment was found to be 2.4% among female healthcare workers and 1.3% among male healthcare workers^(38)^. In a systematic review conducted to determine the prevalence of sexual harassment among healthcare professionals in high-income countries, it was found to be 6.0%^(39)^. The observed differences in findings may be explained by variations in the perception of sexual harassment, cultural contexts, and the structural characteristics of the environments in which the studies were carried out. Due to concerns about stigma or exposure, participants may have withheld information, leading researchers to investigate all forms of violence rather than focusing solely on sexual harassment. Despite varying definitions, sexual harassment appears to be prevalent among healthcare professionals in Türkiye and requires greater attention due to its negative impact on health and quality of life.

In the studies examined, it is observed that the majority of sexual harassment victims are nurses, women, young, single, and newly employed. In a systematic review study, it was reported that female nurses have experienced sexual harassment more than male nurses^(40)^; another study reported that 10.0% to 87.3% of female nurses were exposed to sexual harassment at work^(11)^. In another systematic review and meta-analysis, being younger than 40 years of age and having a marital status of being single, separated, or widowed were reported to be associated with sexual harassment^(41)^. In a study conducted among nurses in Greece, it was found that younger and single nurses were more likely to experience sexual harassment. In contrast, divorced nurses with 1–5 years of professional experience reported more frequent exposure to sexually coercive behaviors^(42)^. In a field dominated by women, nurses are even more susceptible to sexual harassment in the workplace, and sexual harassment against nurses is becoming an occupational threat.

Although the victims of sexual harassment in the studies examined within the scope of this study referred to perpetrators of different groups, it is an important finding that the majority of them mentioned physicians. In a study conducted in Greece to identify sexual harassment among nurses, male physicians were found to be the most frequently reported perpetrators^(42)^. In another study conducted in Ghana examining sexual harassment among nurses, male physicians were identified as the main perpetrators of sexual harassment^(43)^. According to the WHO, the perpetrators of harassment and violence may be people in positions of authority who are respected and trusted, such as physicians^(44)^. It is also noteworthy that most perpetrators were patients^(10,12,45)^ and patient relatives^(43,46)^ in other studies. The majority of the samples in the studies included in this review consisted of nurses, and it can be assumed that nurses are more vulnerable due to power differentials within healthcare institutions, where physicians hold higher hierarchical positions. In addition, nurses may be more exposed to sexual harassment as they have more frequent contact and communication with other healthcare professionals, patients, and patients’ relatives, and they spend longer hours in the hospital setting.

The reviewed studies revealed that healthcare workers faced sexual harassment most often in patient rooms, non-clinical areas, inpatient clinics, and during night shifts. One study found most harassment occurred during night shifts (18:00–07:00)^(47)^, while another reported daytime incidents(46). Similarly, a study conducted among healthcare workers in Saudi Arabia reported that night shift workers (18:00–07:00) were significantly more exposed to sexual violence^(48)^. In Greece, physicians and nurses working in emergency departments and outpatient clinics were 2.696 times more likely to report sexual harassment compared with those working in other departments^(49)^. Working alone with patients increases risks for healthcare workers, particularly nurses in clinical units. Preventive systems are needed to ensure safer workplaces for female staff in healthcare organizations.

It is also noteworthy that healthcare professionals do nothing or ignore the incident. Participants stated that they did not make any attempts since they were afraid of the consequences, ashamed, and felt guilty. Studies indicate that many victims, particularly nurses and physicians, refrain from reporting sexual harassment due to fear of consequences, shame, guilt, or a belief that no action will be taken^(12,46,50)^. Lu et al.^(36)^ stated in their meta-analysis that healthcare professionals in Egypt and Türkiye remained passive against sexual harassment, and female nurses in particular did not report sexual harassment incidents since they were embarrassed. In a qualitative study conducted in Iran examining nurses’ silence in response to sexual harassment, four subcategories were identified, namely fear of social stigma, organizational and legal barriers, family-related barriers, and personal barriers^(51)^. In a study, it was found that approximately one in three nurses did not report sexual harassment due to fear of negative consequences and the belief that no action would be taken against the perpetrator^(42)^. Sexual harassment is a very sensitive issue in conservative communities, and it can be seen as a shameful situation to talk about. In addition, as mentioned in the studies, the fact that the perpetrators were also colleagues or people in a supervisory position may cause victims to hide the incident of sexual harassment.

Sexual harassment has many consequences for healthcare professionals. In the studies considered within the scope of this systematic review, these consequences were usually considered physical and emotional/psychological; however, it was also emphasized that there were important organizational consequences such as decreased productivity, thoughts of leaving the job, and changing workplaces. In the literature, the consequences of sexual violence also include psychological stress, shame, depression, sleep disorders, impaired practice, and unhealthy and uneasy relationships with patients^(52,53)^. Lu et al.^(36)^ found that sexual harassment increased stress on nurses, while reducing job satisfaction, job quality, and productivity. Chakraborty et al.^(50)^ stated that violent incidents reduced job satisfaction in healthcare professionals. A study conducted among allied healthcare personnel and students in the United States found a positive correlation between sexual harassment and burnout^(54)^. It can be concluded that the findings of this study are compatible with the literature.

## CONCLUSIONS

In this study, sexual harassment incidents experienced by healthcare professionals in Türkiye were presented using the systematic review method. As a result of the screening, 16 studies that met the criteria were examined. It was found that the studies were usually conducted in certain professional groups in a single city and institution. All of the studies were cross-sectional descriptive studies, usually using questionnaires developed by the researchers. Accordingly, it can be stated that the causal relationship between the harassment incident and the reactions of the healthcare professional who was subjected to harassment or how sexual harassment incidents change over time has not been examined. In addition, some of the studies only examined sexual harassment, while some examined sexual harassment in a limited perspective along with different types of violence. Although there were healthcare professionals who had never been subjected to sexual harassment as a result of the studies examined, there were also studies reporting high sexual harassment rates. In addition, an equal number of studies focused on all healthcare professionals and nurses, while few addressed physicians exclusively. There was no study examining the incidence of sexual harassment in a single healthcare professional group other than physicians and nurses. The studies revealed that healthcare professionals frequently experienced both verbal and physical sexual harassment, often by multiple perpetrators including colleagues, patients, and their relatives. Victims generally remained passive, underreported incidents, and suffered adverse physical, psychological, and occupational outcomes.

More comprehensive and multi-methodological research is needed to develop a holistic model explaining the characteristics of sexual harassment incidents experienced by healthcare workers in Türkiye, the responses developed against harassment, coping strategies, and the changes in these responses over time. The fact that the current literature is largely limited to descriptive studies makes it difficult to reveal the dynamic and contextual dimensions of the phenomenon adequately. Therefore, in addition to quantitative studies that allow comparisons between different healthcare professional groups, types of institutions, cities, and levels of healthcare services, phenomenological and other qualitative research approaches should be used to ensure a deeper understanding of experiences. Such studies will make significant contributions to understanding the multi-dimensional nature of sexual harassment and developing intervention models.

On the other hand, to prevent and effectively manage sexual harassment against healthcare workers, it is crucial to develop clear, comprehensive, and binding national policies, establish national data collection systems that allow for regular monitoring of the prevalence of harassment, and adopt zero-tolerance approaches supported by legal regulations. Furthermore, it is recommended that training programs be implemented in both educational institutions and healthcare organizations to raise awareness about sexual harassment among healthcare professionals, patients, and their relatives. At the healthcare institution level, it is suggested that the issue of sexual harassment be addressed within the scope of occupational health and safety studies, accessible and secure reporting mechanisms be established, a supportive work environment be provided for victims, legal processes be implemented quickly and effectively in response to incidents, and training be organized for managers and employees to increase institutional awareness. It is believed that these measures will contribute to ensuring accountability and increasing deterrence regarding sexual harassment.

## Data Availability

The entire dataset supporting the results of this study was published in the article itself.
