# Supplementary material for: A situation ignored in the healthcare: a systematic review of sexual harassment against healthcare professionals
Source: Rev Esc Enferm USP. 2026 Oct 12;60:e20250419. doi: 10.1590/1980-220X-REEUSP-2025-0419en (PMC13660675; doi:10.1590/1980-220X-REEUSP-2025-0419en)
Supplement: Translation

# Translation: Una situación ignorada en el ámbito sanitario: revisión sistemática sobre el acoso sexual contra profesionales de la salud

**DOI:** 10.1590/1980-220X-REEUSP-2025-0419es

**Authors:** Sinem Saricoban, Handan Alan, Hatice Ulusoy

**Affiliations:** 1 Sivas Cumhuriyet University, Faculty of Health Sciences, Department of Health Management, Sivas, Turquia.; 2 Istanbul University-Cerrahpaşa, Florence Nightingale Nursing Faculty, Department of Nursing Management, Istanbul, Turquia.

**Keywords:** Acoso Sexual, Personal de Salud, Violencia Laboral, Enfermeras y Enfermeros

## Abstract

**Objetivo:**

El objetivo de este estudio fue examinar sistemáticamente los hallazgos de investigaciones previas realizadas en Turquía sobre el acoso sexual contra profesionales de la salud y explorar sus experiencias de acoso sexual.

**Método:**

El estudio se llevó a cabo mediante el método de revisión sistemática, siguiendo las directrices del protocolo PRISMA. Se realizaron búsquedas utilizando diversas palabras clave en bases de datos nacionales e internacionales, y se incluyeron 16 estudios en la revisión.

**Resultados:**

La tasa de exposición al acoso sexual varía entre el 0,0% y el 75,0%. Las mujeres profesionales de la salud, el personal de enfermería, las personas de entre 28 y 34 años y quienes trabajan en servicios de hospitalización tienen mayor probabilidad de sufrir acoso sexual. Los profesionales de la salud señalaron con mayor frecuencia a los médicos como acosadores. Se ha constatado que los profesionales de la salud víctimas de acoso sexual a menudo no hacían nada al respecto e ignoraban dicho comportamiento.

**Conclusión:**

Se necesitan más investigaciones en profundidad para comprender las experiencias y las estrategias de afrontamiento de los profesionales de la salud que sufren acoso sexual en Turquía, así como el impacto de la cultura en ellos. Las normas de género, la estigmatización y las jerarquías en el lugar de trabajo suelen impedir la denuncia. Los responsables de las políticas sanitarias deberían desarrollar estrategias para abordar estas barreras.

## INTRODUCCIÓN

La violencia es un problema significativo de salud pública que afecta la vida de miles de millones de personas cada año a través de la muerte, las lesiones o las enfermedades^(1)^. Con el aumento global de los incidentes violentos, esta afecta a personas de diversos estratos sociales y puede surgir en distintos entornos, incluyendo los hogares, los espacios públicos, las instituciones educativas, los lugares de trabajo y otras instituciones. La violencia en el lugar de trabajo se ha convertido en una tendencia preocupante entre los trabajadores de diferentes profesiones y entornos laborales en todo el mundo^(2)^. La violencia en el lugar de trabajo puede definirse como «cualquier acto en el que una persona sea maltratada, amenazada, intimidada o agredida en el marco de su empleo»^(3)^. La Administración de Seguridad y Salud Ocupacional (OSHA) define la violencia en el lugar de trabajo como «cualquier acto o amenaza de violencia contra los trabajadores que pueda ocurrir dentro o fuera del lugar de trabajo y que abarca desde amenazas y abuso verbal hasta agresiones físicas y homicidio»^(4)^.

La violencia en el lugar de trabajo es un fenómeno que afecta a casi todos los sectores y categorías de trabajadores; sin embargo, ciertos grupos ocupacionales están expuestos de manera desproporcionada a un mayor riesgo de sufrir este tipo de violencia^(2,5)^. En particular, la violencia que ocurre en entornos de atención médica constituye una preocupación significativa para la salud pública y representa un problema de salud ocupacional cada vez más importante para los trabajadores de la salud^(2,3)^.

En los últimos diez años, se ha registrado un notable aumento de la violencia contra los profesionales de la salud a nivel mundial^(6)^. Un estudio que investigó la violencia contra los profesionales de la salud, utilizando un enfoque de revisión global y metaanálisis, reveló que la tasa general de violencia entre los profesionales de la salud se situó en el 58,7%, lo que comprende violencia física en un 20,8%, violencia verbal en un 66,8% y acoso sexual en un 10,5%^(7)^. Si bien el acoso sexual es menos común en comparación con otras formas de violencia, sigue siendo un problema frecuente entre los profesionales de la salud y tiene efectos negativos. El acoso sexual es una forma de acoso que puede incluir solicitudes de favores sexuales, comentarios ofensivos o conductas sexuales físicas^(8)^. Una revisión paraguas de revisiones sistemáticas y metaanálisis para evaluar la violencia en el lugar de trabajo contra los trabajadores de la salud reportó tasas de acoso sexual entre el 6% y el 36,2%^(9)^. De manera similar, otra revisión sistemática que examinó el acoso sexual en enfermeras encontró tasas que oscilaban entre el 10% y el 87,3%^10^. El acoso sexual es un fenómeno multifacético que abarca conductas verbales, visuales, no verbales o físicas no deseadas de naturaleza sexual o basadas en el sexo de una persona. El acoso sexual físico incluye conductas no consentidas, como toques no deseados, caricias, abrazos y besos.

Por el contrario, el acoso sexual verbal puede manifestarse en forma de comentarios sexualmente sugerentes, comentarios ofensivos o chistes sexualmente explícitos. El acoso sexual no verbal abarca comportamientos como miradas intimidantes o persistentes, gestos y expresiones faciales inapropiados, así como la exhibición o transmisión de fotografías, imágenes o regalos sexualmente explícitos. Además, con el uso generalizado de las tecnologías de la información y la comunicación, el acoso sexual cibernético —incluido el envío de correos electrónicos o mensajes de texto no deseados, ofensivos y sexualmente explícitos— se ha convertido en una forma cada vez más frecuente de acoso sexual^(11)^.

Los estudios que investigan el acoso sexual entre los profesionales de la salud indican que las personas sometidas a acoso sexual suelen reportar síntomas físicos, psicológicos y laborales negativos tras dichos incidentes^(12-14)^.

En la literatura internacional se pueden encontrar estudios sistemáticos y metaanálisis que examinan el acoso sexual contra los profesionales de la salud^(10,15)^. Sin embargo, no se ha encontrado ningún estudio sistemático sobre este tema en Turquía. Si bien las normas patriarcales y los roles de género tradicionales aumentan el riesgo de acoso sexual, las enfermeras y otras trabajadoras de la salud son particularmente vulnerables al acoso perpetrado por pacientes, familiares de pacientes e incluso colegas. Además, las barreras socioculturales y legales, como la estigmatización, las actitudes que culpan a la víctima y la naturaleza onerosa de los procedimientos legales, pueden disuadir a las víctimas de denunciar dichos incidentes ante las autoridades oficiales^(16,17)^. Los factores estructurales y organizativos dentro del sistema de salud, entre ellos la elevada carga de pacientes, las largas jornadas laborales y las medidas de seguridad inadecuadas (especialmente durante los turnos nocturnos), agravan los riesgos estructurales existentes al hacer que el personal sea más vulnerable frente a los pacientes y sus familiares^(18)^. Teniendo en cuenta todas estas razones, este estudio investiga el papel de los determinantes culturales y contextuales específicos de Turquía en la configuración de la prevalencia, el riesgo y los factores de protección del acoso sexual entre los trabajadores de la salud, centrándose en los incidentes de acoso sexual vividos por los trabajadores de la salud en Turquía, en lugar de en los extensos estudios sobre acoso sexual presentes en la literatura mundial. Se espera que los hallazgos mejoren la comprensión de las realidades locales, contribuyan a las interpretaciones interculturales del acoso sexual y sirvan de base para el desarrollo de políticas de salud y estrategias de prevención específicas para cada contexto. En esta revisión sistemática, el objetivo fue examinar de manera sistemática los hallazgos de estudios previos realizados en Turquía relacionados con el acoso sexual contra los profesionales de la salud. También se buscó conocer las experiencias de acoso sexual de los profesionales de la salud.

## MÉTOD

### Tipo de Estudio, Protocolo y Registro

Este estudio se llevó a cabo utilizando el método de revisión sistemática y siguió el protocolo del Instituto Joanna Briggs (JBI)^(19)^. Se siguió la guía «Elementos preferidos para el reporte de revisiones sistemáticas y metaanálisis» (PRISMA) en la elaboración del protocolo del estudio y la redacción del informe^(20)^. El protocolo del estudio se registró en PROSPERO (número de registro: CRD42023421654).

### Preguntas de la Revisión

Esta revisión sistemática buscó esclarecer las siguientes preguntas de investigación:

En cuanto a los profesionales de la salud:

- ¿Cuáles son los hallazgos respecto al acoso sexual al que estuvieron expuestos?
- ¿Qué hallazgos se han reportado a raíz del acoso sexual?
- ¿Cuáles son las recomendaciones para la prevención del acoso sexual?

La pregunta de la revisión está estructurada según el marco PCC (Población–Concepto–Contexto). En consecuencia, la población incluye a todos los trabajadores de la salud, tales como médicos, enfermeras, parteras y técnicos de la salud que trabajan en Turquía; el concepto abarca todos los tipos de acoso sexual, incluyendo el acoso verbal, físico, psicológico y el acoso laboral (mobbing); y el contexto cubre todas las instituciones de salud en Turquía.

### Criterios de Elegibilidad

Esta revisión sistemática examinó artículos de investigación que presentaran hallazgos relacionados con el acoso sexual entre los trabajadores de la salud en Turquía. El estudio incluyó todas las formas de violencia, sin importar el tipo; también se incluyeron estudios que abordaran el acoso sexual junto con otros tipos de violencia. Se incluyeron en el alcance del estudio los artículos de investigación originales publicados en turco o en inglés, cuyo texto completo estuviera disponible. No se aplicaron restricciones en cuanto al año de publicación, el tipo de acoso sexual, el grupo ocupacional o el tipo de institución de salud. En el marco de los criterios de exclusión, se han excluido de la evaluación las publicaciones cuyo texto completo no esté disponible, los estudios publicados en idiomas distintos del turco o el inglés, las ponencias de congresos, las notas, las reseñas de congresos, los capítulos de libros, los editoriales, las encuestas breves, las cartas y los artículos de revisión.

### Estrategia de Búsqueda

En el marco del estudio, se indexaron los trabajos de investigación publicados hasta el 31 de diciembre de 2023 utilizando cinco bases de datos nacionales e internacionales con palabras clave en inglés, de acuerdo con los «Medical Subject Headings (MESH)», y palabras clave en turco, de acuerdo con los Términos Científicos Turcos, en PubMed, ScienceDirect, Emerald, Web of Science y DergiPark» (Tabla 1). Las palabras clave se buscaron combinándolas con el operador «AND» en cada base de datos. En la base de datos turca DergiPark usamos: Las combinaciones de palabras clave “Cinsel Taciz AND Doktor AND Türkiye”, “Cinsel Taciz AND Hekim AND Türkiye”, “Cinsel Taciz AND Hemşire AND Türkiye”, “Cinsel Taciz AND Sağlık Personeli AND Türkiye”, “Cinsel Taciz AND Sağlık Çalışanı AND Türkiye. Una vez más, se realizaron búsquedas en inglés utilizando la base de datos DergiPark, con sede en Turquía, y otras bases de datos como PubMed, Science Direct, Emerald y Web of Science, con los siguientes términos de búsqueda: “Sexual Harassment AND Doctors AND Turkey”, “Sexual Harassment AND Physicians AND Turkey”, “Sexual Harassment AND Nurses AND Turkey”, “Sexual Harassment AND Health Staff AND Turkey”, “Sexual Harassment AND Healthcare Staff AND Turkey”, “Sexual Harassment AND Health Care Staff AND Turkey”, “Sexual Harassment AND Health Professionals AND Turkey”, “Sexual Harassment AND Healthcare Professionals AND Turkey”, “Sexual Harassment AND Health Care Professionals AND Turkey”, “Sexual Harassment AND Health Workforce AND Turkey”, “Sexual Harassment AND Healthcare Workforce AND Turkey”, “Sexual Harassment AND Health Care Workforce AND Turkey”, “Sexual Harassment AND Health Personnel AND Turkey”, “Sexual Harassment AND Healthcare Personnel AND Turkey”, “Sexual Harassment AND Health Care Personnel AND Turkey”, “Sexual Harassment AND Health Workers AND Turkey”, “Sexual Harassment AND Healthcare Workers AND Turkey”, “Sexual Harassment AND Health Care Workers AND Turkey”.

**Tabla 1:** Bases de datos y palabras clave – Turquía, 2024.

Bases de datos	Palabras clave en turco	Palabras clave en inglés
DergiPark	Cinsel taciz, doktor, hekim, hemşire, sağlık personeli, sağlık çalışanı, Türkiye	
PubMed, Science Direct, Emerald, Web of Science		Sexual harassment, doctors, physicians, nurses, health staff, healthcare staff, health care staff, health professionals, healthcare professionals, health care professionals, health workforce, healthcare workforce, health care workforce, health personnel, healthcare personnel, health care personnel, health workers, healthcare workers, health care workers, Turkey

### Proceso de Selección

Se utilizó el programa EndNote X9, destinado a la gestión de información bibliográfica, para recolectar los estudios de las bases de datos. En EndNote, se creó una biblioteca para cada base de datos; posteriormente, se combinaron las bibliotecas y se excluyeron los estudios duplicados. El proceso de selección fue realizado de manera simultánea e independiente por dos autores (SS, HA); se examinaron los títulos y los resúmenes de los estudios, y los autores compararon todos los estudios resultantes. En la siguiente etapa, todos los autores evaluaron los textos completos de los estudios de acuerdo con los criterios de inclusión y exclusión. En la última etapa, se incluyeron 16 artículos en la revisión sistemática. El proceso se describió en detalle en el diagrama de flujo PRISMA (Figura 1).

### Evaluación de la Calidad Metodológica de los Estudios

La evaluación de la calidad de los estudios seleccionados se realizó utilizando la Lista de verificación analítica transversal del JBI. La lista de verificación consta de 8 preguntas, y cada pregunta se evalúa como «sí», «no», «poco claro» o «no aplicable». Dos investigadores (SS, HA) realizaron las evaluaciones de manera independiente, y un tercer investigador (HU) resolvió las discrepancias. En la evaluación de calidad, se consideró como puntaje de calidad (PC) el número de ítems calificados como «sí» de un total de ocho.

### Revisión de la Literatura

Para reducir el riesgo de posibles sesgos durante el estudio, la revisión bibliográfica, la selección de artículos, la recolección de datos y los procedimientos de evaluación de la calidad de los artículos fueron realizados por dos investigadores (SS y HA) de manera independiente, y cada etapa del proceso fue revisada por un tercer investigador (HU), tras lo cual se llegó a un consenso. Además, tres investigadores (SS, HA, HU) participaron en un estudio piloto para llevar a cabo estas operaciones de manera correcta y exitosa. Este estudio piloto consistió en revisar los artículos, recopilar datos y evaluar su calidad. Las diferencias de opinión que surgieron tras el estudio piloto se resolvieron mediante discusión.

### Recolección de Datos

Para obtener los datos del estudio se utilizó una herramienta de recolección de datos desarrollada por los investigadores. Con esta herramienta se obtuvieron datos relacionados con los autores y el año de publicación, el lugar donde se llevó a cabo el estudio, las características de los participantes y la situación de acoso sexual.

### Aprobación Ética

No se requirió aprobación ética, ya que los datos eran datos secundarios obtenidos de las bases de datos. Este estudio no implicó experimentos en seres humanos ni en animales, y no se realizó la recolección de datos de los participantes a nivel individual.

### Análisis de Datos

La metodología, las características de los participantes, los métodos de evaluación y las variables de resultado de los estudios incluidos en la revisión sistemática varían en consecuencia. Por lo tanto, los hallazgos se tabularon y se presentaron en forma narrativa, ya que no se pudo realizar un metaanálisis.

### Limitaciones

El hecho de que la gran mayoría de los estudios examinados tengan un diseño transversal y que los datos se recolecten mediante autoinformes limita la inferencia causal y aumenta el riesgo de sesgo de respuesta. La falta de una clasificación estandarizada de los tipos de acoso sexual y de los grupos ocupacionales ha dado lugar a una heterogeneidad metodológica significativa entre los estudios, lo que dificulta la comparación directa de las estimaciones de prevalencia. Además, el hecho de no haber utilizado herramientas de medición validadas y confiables en todos los estudios, así como la preferencia por cuestionarios no estandarizados desarrollados por los propios investigadores en algunos de ellos, ha aumentado el riesgo de error de medición y de sesgo sistemático. El proceso de recolección de datos basado en el autoinforme es susceptible al sesgo de recuerdo, dependiendo de cómo los participantes recuerden sus experiencias pasadas, y puede afectar la precisión de las tasas de prevalencia reportadas. Sin embargo, el hecho de que una parte significativa de los estudios se haya llevado a cabo en Ankara (la capital de Turquía) indica una concentración regional; la ausencia de muestras representativas a nivel nacional limita la generalizabilidad de los hallazgos a Turquía en su conjunto.

Además, dado que el acoso sexual es un fenómeno estigmatizante, los participantes pueden sentirse inclinados a subestimar o ocultar sus experiencias. Por lo tanto, las tasas de prevalencia reportadas pueden subestimar la situación real. Por último, el hecho de que los estudios se hayan realizado predominantemente en instituciones de salud de nivel secundario y terciario, junto con la falta de investigaciones que abarquen los servicios de salud primaria y el sector de salud privado, es otro factor importante que limita la generalizabilidad de los hallazgos.

## RESULTADOS

### Resultados de la Selección

Las búsquedas iniciales en las bases de datos identificaron 5 169 artículos. Tras eliminar los duplicados en EndNote X9, quedaron 890 registros. La selección de títulos y resúmenes redujo esta cifra a 91 artículos para la revisión del texto completo. De estos, 16 cumplieron con los criterios de inclusión al informar sobre las experiencias de acoso sexual de los profesionales de la salud (Figura 1). Estos 16 estudios se incluyeron en el análisis final.

**Figura 1:** Flujograma PRISMA – Turquía, 2024.

### Características Generales de los Estudios

Los estudios incluidos en la revisión se realizaron entre 1996 y 2023; 15 fueron estudios cuantitativos descriptivos, mientras que en un estudio^(18)^ se utilizaron métodos cuantitativos y cualitativos descriptivos. Mientras que dos estudios^(21,22)^ se llevaron a cabo en toda Turquía, cinco se realizaron en Ankara^(23-27)^, y en cinco estudios no se especificó la ciudad o región^(18,28-31)^. Cada uno de los estudios restantes se llevó a cabo en ciudades diferentes^(32-35)^.

Al analizar las instituciones donde se llevaron a cabo los estudios, se observó que ocho se realizaron en hospitales públicos^(23,24,27,30,31,33-35)^, ocho en hospitales de formación e investigación^(25,27,29-33,35)^ y dos en hospitales privados^(31-33)^. En cuatro artículos^(18,22,26,28)^ no se especificaron las instituciones donde se llevaron a cabo los estudios. En un estudio, se indicó que la investigación se realizó en instituciones de salud de atención primaria, secundaria y terciaria^(21)^.

Se realizaron un total de 11 estudios en todo el hospital^(18,21,23-26,28-30,34,35)^, cuatro en servicios de urgencias^(22,27,32,33)^ y uno en el quirófano^(31)^.

Ocho de los estudios se realizaron con enfermeras^(23,25,26,29-31,34,35)^, seis se realizaron con todos los profesionales de la salud^(21,24,27,28,32,33)^ y dos se realizaron con médicos^(18,22)^. El tamaño total de la muestra de los estudios fue de 18 014 profesionales de la salud, y el tamaño de la muestra de cada estudio osciló entre 49 y 12 944.

En seis de los estudios, solo se investigó el acoso sexual en los profesionales de la salud^(18,23,25,26,30,35)^, mientras que en 10 estudios se consideró junto con otros tipos de violencia^(21,22,24,27-29,31-34)^ (Tabla 2).

**Tabla 2:** Características bibliográficas de las investigaciones (N = 16) – Turquía, 2024.

Variable		n
Tipo de estudio	Cuantitativo	15
	Cuantitativo + Cualitativo	1
Lugar (ciudad) donde se llevó a cabo el estudio*	Ankara	5
	En toda Turquía	2
	Sin especificar	5
	Otro	4
Institución donde se llevó a cabo el estudio*	Hospital de formación e investigación	8
	Hospital público	8
	Hospital privado	2
	Sin especificar	4
Unidad donde se llevó a cabo el estudio	Hospital	11
	Servicio de urgencias	4
	Sala de operaciones	1
Grupo de muestra	Enfermera	8
	Todos los profesionales de la salud	6
	Médico	2
Número de muestras	≤200	6
	≥201	10
Alcance de la investigación	Acoso sexual/violencia sexual	6
	Todos los tipos de violencia	10

*Algunos de los estudios se llevaron a cabo en más de una ciudad e institución.

### Herramientas de Recolección de Datos Utilizadas en los Estudios

En todos los estudios se utilizaron cuestionarios como herramienta de recolección de datos. En tres estudios se emplearon diferentes escalas desarrolladas en otros estudios^(24,26,29)^. En tres estudios se utilizaron formularios creados a partir de cuestionarios publicados por organizaciones como la Organización Mundial de la Salud (OMS), la Organización Internacional del Trabajo (OIT), la Asociación Internacional de Enfermeras (ICN) y la Asociación Médica de Estados Unidos (AMA) se utilizaron en tres estudios^(21,28,34)^, mientras que el cuestionario creado por los investigadores a partir de estudios similares en la literatura se utilizó en 10 estudios^(18,22,23,25,27,30-33,35)^ (Tabla 3).

**Tabla 3:** Características de los estudios sobre el acoso sexual contra los profesionales sanitarios en Turquía – Turquía, 2024.

N.º del estudio	Autor(es), año y QS	Lugar del estudio	Diseño de la investigación	Muestra	Herramientas de recopilación de datos	Principales conclusiones
1	Kisa y Dziegielewski 1996^(25)^, QS:6/8	1 hospital universitario	Descriptivo	184 enfermeras	Cuestionario de Cho-lewinski y Burge (1990) cuestionario elaborado a partir del Inventario de Acoso Sexual y del cuestionario de Phillips y Schneider (1993), (18 preguntas de opción múltiple)	El 75% de las enfermeras declaró haber sido víctima de acoso sexual. Los tipos más comunes fueron las bromas de carácter sexual (72%), los gestos obscenos (65%) y la coacción para conseguir una cita (53%). Los acosadores eran en su mayoría médicos (43%) y pacientes (34%). El 39% sufrió acoso entre 1 y 4 veces a lo largo de su carrera, y el 41% de los casos se produjo durante los turnos de noche (16:00–12:00). Entre las respuestas más habituales se encontraban ignorar el comportamiento (29%), decirle a la persona que dejara de hacerlo (26%), bromear (18%) y evitar la situación (18%). El acoso se produjo principalmente en las habitaciones de los pacientes (32%), en áreas no clínicas (30%) y en las salas del hospital (20%). El 47% declaró haber sufrido una disminución de la productividad. Las reacciones emocionales incluyeron ira (44%), humillación (14%) y miedo (12%), mientras que los síntomas físicos fueron dolores de cabeza (38%), insomnio (20%) y fatiga (15%).
2	Kisa et al. 2002^(30)^, QS:6/8	1 universidad y 1 hospital público	Descriptivo	215 enfermeras	El cuestionario elaborado por Kısa y Dziegielewski (1996) (18 preguntas de opción múltiple)	El 73% de las enfermeras declaró haber sufrido acoso sexual. Entre los tipos más comunes se encontraban las miradas insinuantes (20%), las bromas de carácter sexual (19%) y los gestos obscenos (18%). Los acosadores eran, en su mayoría, médicos (41%) y pacientes (39%). El 38,4% de los incidentes tuvo lugar durante los turnos de noche. El acoso se produjo principalmente en las habitaciones de los pacientes (34%), en áreas no clínicas (30%) y en los servicios hospitalarios (18%). Las enfermeras solían responder ignorando el comportamiento (29%), evitando al acosador (21%) o bromeando al respecto (15%). El 45% declaró haber experimentado una disminución de la productividad. Las reacciones emocionales incluyeron ira (42,9%), miedo (14,1%) y humillación (10,5%). Los síntomas físicos fueron dolor de cabeza (37,7%), insomnio (20,2%) y gastritis (12,9%).
3	Ayrancı 2005^(33)^, QS:6/8	Servicios de urgencias	Descriptivo	195 profesionales sanitarios	Cuestionario elaborado por los investigadores a partir de la bibliografía	El personal de los servicios de urgencias que participó en el estudio afirmó que nunca había sido objeto de acoso sexual anteriormente.
4	Çelik y Çelik, 2007^(23)^, QS:8/8	8 hospitales públicos	Descriptivo	622 enfermeras	Un cuestionario elaborado por los investigadores a partir de la bibliografía y de la Asociación Médica Americana	La prevalencia del acoso sexual entre el personal de enfermería es del 37,1%. Se registraron tasas más elevadas entre el personal de enfermería soltero (42,3%), los recién titulados en enfermería (52,8%), las personas de entre 30 y 34 años (45,7%), con entre 10 y 14 años de experiencia (47,7%) y quienes trabajan en unidades de hospitalización (39,6%). Los acosadores eran principalmente médicos (77,1%), otras enfermeras (51,1%) y pacientes (43,3%). La forma más habitual fue la de chistes, historias o comentarios sexuales no deseados. El 45% declaró haber experimentado una disminución de la productividad; el 37,2% se planteó dejar el trabajo; el 36,8% señaló que su vida social se había visto afectada, y el 27,3% que su vida familiar; el 44,6% sufrió problemas de salud mental, el 40,3% dolores de cabeza y el 24,2% problemas de salud física. Entre las reacciones más habituales se encontraban no hacer nada (59,3%), establecer límites (43,3%) e ignorar el incidente (30,7%).
5	Erdemir et al. 2011^(35)^, QS: 7/8	1 hospital universitario, 2 hospitales públicos	Descriptivo (cuantitativo y cualitativo)	184 enfermeras	Cuestionario elaborado por los investigadores a partir de la bibliografía (13 preguntas)	El 71,7% de las enfermeras declaró haber sufrido acoso sexual por parte de los pacientes. Las formas más comunes fueron miradas incómodas (61,4%), preguntas personales intrusivas (34,8%) y amenazas sexuales o lenguaje soez (26,1%). Tras el incidente, el 31,4% actuó como si nada hubiera pasado, el 29,3% buscó ayuda de compañeros o superiores y el 18,5% rechazó directamente el comportamiento. Las sugerencias de las enfermeras para la prevención incluían la autorrestricción, limitar la comunicación con los pacientes, mejorar la seguridad y la supervisión, impartir formación y sensibilización, y modificar o interrumpir la atención al paciente.
6	Talas et al. 2011^(27)^, QS:6/8	Servicios de urgencias	Descriptivo (cuantitativo y cualitativo)	270 miembros del personal de los servicios de emergencia	El cuestionario elaborado por los investigadores a partir de la bibliografía (El cuestionario de 36 preguntas —28 de respuesta cerrada y 8 de respuesta abierta—)	El 15,9% de los profesionales sanitarios declaró haber sufrido acoso sexual. Se observaron tasas más elevadas entre las mujeres, el personal de enfermería, los titulados universitarios y las personas de entre 28 y 34 años. Las formas más comunes incluían chistes o comentarios sexuales no deseados (51,2%), gestos sugerentes (37,2%) y propuestas de cita no deseadas (39,5%). Los acosadores eran, en su mayoría, familiares de los pacientes, pacientes, médicos y personal de enfermería. Las reacciones incluyeron no hacer nada (37,2%), abandonar el lugar (37,2%) y denunciar el incidente (34,9%). Las respuestas emocionales más comunes fueron la tristeza, la ira y la frustración.
7	Ulusoy et al. 2011^(18)^, QS:6/8	Un hospital de 1000 camas	Descriptivo (cuantitativo y cualitativo)	49 médicos adjuntos	El cuestionario elaborado por los investigadores (12 preguntas de tipo «Sí/No»)	El 67,3% de las médicas adjuntas declararon haber sufrido acoso sexual. Las formas más comunes fueron las miradas incómodas (51,0%), las preguntas de carácter sexual explícito y las invitaciones a salir (ambas con un 14,3%). Las estrategias de afrontamiento incluían solicitar el alta del paciente (24,2%), romper el contacto con el acosador (24,2%), rechazar el comportamiento (21,2%), ignorar el incidente (21,2%) y buscar ayuda (12,1%). Las medidas de prevención se centraban en la interacción entre el médico y el paciente, la apariencia, evitar estar a solas con los pacientes y utilizar barreras físicas entre los médicos y los pacientes o sus familiares.
8	Tekin y Bulut 2014^(31)^, QS: 8/8	17 hospitales públicos, 8 universitarios y 13 privados	Estudio descriptivo	380 enfermeras quirúrgicas	Cuestionario elaborado por los investigadores a partir de la bibliografía (48 preguntas)	El 32,6% de las enfermeras de quirófano declararon haber sufrido acoso sexual, principalmente por parte de médicos (94,4%), compañeros de trabajo (41,1%) y otro personal (20,2%). Las formas más comunes fueron bromas o comentarios no deseados (89,5%), gestos sugerentes (52,4%) y presión para mantener relaciones fuera del ámbito laboral (26,6%). Las reacciones emocionales incluyeron ira (50%), sorpresa (28,2%) y humillación (25%). Mientras que el 40,3% restó importancia al incidente, otras personas sintieron que podían sobrellevarlo (22,6%), se culparon a sí mismas (11,3%) o creyeron que no se lo merecían (5,6%). Las reacciones posteriores al incidente incluyeron ignorarlo (40,3%), enfrentarse al acosador (21%), hablar del tema (16,1%), cortar la comunicación (10,5%) y denunciarlo a los responsables.
9	Pınar et al. 2017^(21)^ Calidad de la investigación: 8/8	Profesionales sanitarios de atención primaria, secundaria y terciaria	Descriptivo	12 944 profesionales sanitarios	Estudios de casos por países sobre la violencia en el lugar de trabajo en el sector sanitario. Herramientas de investigación: cuestionario elaborado por la OIT, el CIE, la OMS y la ISP (130 preguntas)	El 1% (n = 130) de los profesionales sanitarios declaró haber sufrido acoso sexual, incluidos 11 de atención primaria y 119 de atención secundaria. De ellos, 28 eran hombres y 102 mujeres; 71 eran enfermeros/parteras, 19 médicos/dentistas, 16 miembros del personal de servicios médicos de urgencia, 16 técnicos y el resto desempeñaba diversas funciones. 110 trabajaban en el sector público y 19 en el sector privado. El acoso procedía principalmente de pacientes (38,2%), familiares de pacientes (36%) y compañeros de trabajo (30,8%). Los autores eran predominantemente hombres (78,5%).
10	Aydemir et al. 2020^(32)^, QS: 7/8	Servicio de urgencias de una facultad de medicina	Descriptivo	121 profesionales sanitarios	Cuestionario elaborado por los investigadores a partir de la bibliografía (34 preguntas)	La tasa de acoso sexual del personal de urgencias es del 1,8%.
11	Demirci y 13/07/2026 2020^(24)^, QS: 8/8	1 hospital público	Descriptivo	347 profesionales sanitarios	El cuestionario elaborado por Çelik y şenol-Çelik (2007) (42 preguntas)	La tasa global de acoso sexual entre los profesionales sanitarios es del 16,4%, con tasas más elevadas entre el personal de enfermería (26,9%) en comparación con los médicos (16,3%) y el resto del personal (10,0%). Los médicos fueron los principales acosadores para el 12,5% de los médicos, el 22,6% del personal de enfermería y el 8,0% del resto del personal. El acoso provocó una disminución de la productividad (35,1%) y pensamientos de abandonar el trabajo (21,1%). También afectó a la vida social (22,8%) y familiar (14,0%). Los efectos físicos incluyeron dolores de cabeza (29,8%), deterioro de la salud (22,8%) y dolor abdominal (12,3%); mientras que los efectos psicológicos fueron la ira (64,9%), el deterioro de la salud mental (42,1%) y el miedo (28,1%). Las estrategias más comunes para afrontar la situación fueron no tomar ninguna medida (82,5%), enfrentarse al acosador (52,6%) e ignorar el comportamiento (49,1%). El acoso afectó negativamente a la calidad de la vida laboral de las enfermeras y aumentó su intención de abandonar el puesto.
12	Kırlıoğlu y Toygar, 2020^(26)^ QS: 8/8	En diversos hospitales	Descriptivo	571 enfermeras	La escala desarrollada por Turgut (2007) (22 elementos)	Se ha constatado que la exposición de las enfermeras al acoso sexual afecta negativamente a su calidad de vida laboral y a su intención de abandonar el trabajo.
13	Çam y Üstüner- Top 2021^(34)^, QS:8/8	1 hospital público	Descriptivo	865 enfermeras	La OMS, la OIT, el CIE y la ISP elaboraron el «Cuestionario sobre la violencia en el lugar de trabajo en el sector sanitario»	El acoso sexual representó el 1,7% de todos los actos de violencia sufridos por el personal de enfermería. Diez enfermeras y cinco enfermeros denunciaron haber sido acosados, principalmente por personal del hospital (26,7%), familiares de pacientes (20%) y compañeros de trabajo (20%). La mayoría de los incidentes se produjeron dentro del hospital (60%). Entre las reacciones más comunes figuraban pedir al acosador que dejara de hacerlo (33,3%), informar a amigos o familiares (33,3%) y fingir que no había pasado nada (20%). El 73,3% consideraba que el incidente era inevitable. Entre los motivos para no denunciarlo figuraban el miedo a las consecuencias (40%), la vergüenza (33,3%) y la creencia de que no serviría de nada (26,7%).
14	Dursun y Aytaç 2021^(28)^, QS: 8/8	n/a	Descriptivo	169 profesionales sanitarios	Cuestionario sobre violencia en el lugar de trabajo elaborado por la OMS y la OIT y adaptado por Aytaç et al. (2011)	El 5,4% de los profesionales sanitarios denunció acoso sexual; todas las víctimas eran mujeres. Ningún profesional sanitario varón sufrió acoso. Los acosadores eran en su mayoría hombres (87,5%). Las personas expuestas a la violencia manifestaron una menor satisfacción laboral en comparación con aquellas que no lo estaban.
15	Sabak et al. 2021^(22)^, QS:6/8	Todos los médicos de urgencias afiliados a una asociación	Descriptivo	362 médicos de urgencias	Cuestionario elaborado por los investigadores a partir de la bibliografía (34 preguntas)	La tasa de acoso sexual entre los médicos de urgencias es del 6,1%. Entre las personas afectadas, el 6,7% eran hombres y el 5,1% mujeres. Entre los acosadores se encontraban pacientes (3,3%), familiares (3,3%) y personas en estado de embriaguez (2,2%). El 96,6% señaló la falta de normas hospitalarias que restringieran los movimientos de pacientes y visitantes. Además, el 59,1% señaló la ausencia de personal de seguridad, el 100% afirmó que los guardias no iban armados y el 95,5% se sentía insuficientemente protegido.
16	Erkutlu et al. 2023^(29)^, QS:8/8	5 hospitales universitarios privados y 5 públicos afiliados	Descriptivo	536 enfermeras	La escala desarrollada por Ding et al., 2016 (7 ítems, escala de Likert de seis puntos)	La tasa de acoso sexual entre el personal de enfermería es del 0,4%.

QS: Puntuación de Calidad.

### Hallazgos Sobre la Situación del Acoso Sexual Entre los Profesionales de la Salud

#### Prevalencia y Tipos de Acoso Sexual

En los estudios analizados en esta revisión sistemática, las tasas de acoso sexual entre los trabajadores de la salud oscilaron entre el 0,0% y el 75,0%, y se observó que superaban el 70,0% en tres estudios en los que solo se investigó el acoso sexual^(25,30,35)^. En los estudios realizados junto con otros tipos de violencia, los participantes informaron que lo que más sufrían era abuso verbal o violencia (38,4%–99,0%), mientras que lo que menos sufrían era el acoso sexual (0,0%–32,6%)^(21,22,24,27-29,31-34)^.

Al examinar las muestras, mientras que la tasa de exposición al acoso sexual se reportó entre el 0,0% y el 16,4% en los estudios realizados con todos los profesionales de la salud^(21,24,27,28,32,33)^, se situó entre el 0,4% y el 75,0% en los estudios realizados únicamente con enfermeras^(23,25,29-31,34,35)^, el 67,3% en los estudios realizados con médicas residentes^(18)^ y el 6,1% en los estudios realizados con médicos de urgencias^(22)^. Al analizar algunas características personales y profesionales de los profesionales de la salud que habían sido víctimas de acoso sexual, se observó que las mujeres profesionales de la salud sufrían más acoso sexual en comparación con los hombres^(21,24,27,28,34)^, mientras que, entre los demás profesionales de la salud, las enfermeras eran las más afectadas^(21,24,27)^. Los estudios se realizaron con profesionales de la salud de entre 18 y 51 años y de entre 28 y 34 años^(23,27)^; aquellos que eran solteros, tenían menos experiencia profesional y trabajaban en clínicas de hospitalización presentaban una mayor tasa de exposición al acoso sexual^(23)^.

La frecuencia de la exposición al acoso sexual se reportó en cuatro de los estudios analizados^(23,25,28,30)^ y la proporción de profesionales de la salud que informaron haber sido víctimas de acoso sexual al menos una vez durante su carrera^(25)^ osciló entre el 11,0% y el 77,8%^(28)^.

En el marco de los estudios analizados, la mayoría de los participantes declaró haber sido objeto de acoso sexual verbal, como palabras, chistes e historias con connotaciones sexuales, amenazas sexuales o uso de lenguaje soez, invitaciones a reunirse fuera del trabajo después de la jornada laboral, preguntas sobre la vida privada y llamadas telefónicas o correos electrónicos no solicitados^(18,23,25,27,30,31,35)^.

Además, las participantes también mencionaron conductas de acoso sexual físico, tales como la exposición sexualmente sugerente de ciertas partes del cuerpo, roces, tocamientos, abrazos, agarres, miradas insinuantes, intentos de agresión sexual, miradas fijas e incómodas, intentos de mostrar los genitales, acoso fuera del trabajo, solicitudes de que un profesional de la salud les tocara los genitales e intentos de llevarlas a la cama^(18,23,25,27,30,31,35)^.

### Características de las Víctimas y los Agresores

En los estudios analizados en la revisión sistemática, los participantes informaron que muchas personas los acosaban sexualmente en sus entornos laborales. En cinco de estos estudios, los participantes señalaron que, con mayor frecuencia, eran acosados por médicos^(23-25,30,31)^. Por el contrario, en dos estudios se informó que los agresores eran pacientes^(21,22)^, en un estudio, familiares de pacientes^(27)^, y en otro, personal del hospital^(34)^. Un estudio realizado por Erdemir et al.^(35)^ investigó el acoso sexual sufrido por enfermeras únicamente por parte de pacientes, y un estudio realizado por Ulusoy et al.^(18)^ investigó el acoso sexual sufrido por médicas residentes únicamente por parte de pacientes y familiares de pacientes. Además, también se identificó como acosadores a médicos en residencia, miembros del cuerpo docente, personal de limpieza o mantenimiento, personal de seguridad, otros colegas y directivos^(21,23,25,30,31,34)^. Sabak et al.^(22)^ destacaron que los médicos de urgencias también sufrían acoso sexual por parte de personas ebrias y con enfermedades mentales, además de los grupos mencionados anteriormente.

### Factores Contextuales (Entorno Laboral, Patrones de Turnos, Características Institucionales)

Los participantes en los estudios informaron que el acoso sexual ocurría con mayor frecuencia en las habitaciones de los pacientes, en entornos no clínicos o en clínicas de hospitalización^(25,30,34)^. También indicaron que estaban más expuestos al acoso sexual durante el turno nocturno^(25,30)^.

### Reacciones y Comportamiento Ante el Acoso

En los estudios analizados, los profesionales de la salud que sufrían acoso sexual a menudo no hacían nada^(23,24,27,35)^, actuaban como si nada hubiera pasado e ignoraban el comportamiento^(25,30,31,35)^. Ulusoy et al.^(18)^ determinaron que las médicas residentes preferían que el paciente fuera dado de alta en tales situaciones y cortaban la comunicación. Un porcentaje muy bajo de participantes afirmó haber hablado de la situación con un amigo o un superior cuando fueron víctimas de acoso sexual^(18,23-25,27,30,35)^. Al mismo tiempo, solo un estudio destacó que los profesionales de la salud no contaban ni denunciaban el incidente a nadie más porque temían consecuencias negativas, sentían vergüenza, se sentían culpables y pensaban que no serviría de nada^(34)^.

Los estudios considerados en el marco de la revisión de alcance informaron que las personas se veían afectadas física y emocional/psicológicamente tras sufrir acoso sexual y que estos efectos provocaban consecuencias negativas relacionadas con el trabajo. Los participantes experimentaron físicamente dolores de cabeza, insomnio o dormir más de lo habitual^(18,23-25,27,30,35)^ y, a nivel emocional/psicológico, sintieron ira, humillación, miedo, culpa, autoculpa y se sintieron humillados con frecuencia^(23-25,27,30,31)^.

Como resultado, la productividad y la calidad de vida laboral de los profesionales de la salud disminuyeron, y consideraron renunciar a sus empleos^(23-26,30,31)^.

### Recomendaciones de los Profesionales de la Salud para la Prevención del Acoso Sexual

En dos de los estudios analizados, los profesionales de la salud que sufrieron acoso sexual formularon recomendaciones relacionadas con *(1)* los pacientes y el personal de salud y *(2)* la institución, con el fin de evitar el acoso sexual en el trabajo y prevenirlo^(18,35)^.

(1) *Las recomendaciones relacionadas con los pacientes y los profesionales de la salud* incluyeron dar por terminado el tratamiento y dar de alta al paciente, brindar apoyo psicológico al paciente, no quedarse a solas con el paciente en la habitación, limitar el tiempo asignado al paciente, no establecer contacto visual directo con el paciente o sus familiares, mostrarse firme con el paciente y sus familiares, dirigirse al paciente y a sus familiares de manera profesional, referirse al paciente y a sus familiares como «señor» en lugar de «tío» o «hermano», mantener la comunicación breve, mostrarse serio y no hacer bromas, evitar comportamientos que puedan malinterpretarse, evitar la ropa que llame la atención, preferir ropa discreta y holgada en la medida de lo posible y no usar maquillaje excesivo, mantener la distancia física con los pacientes y sus familiares tanto como sea posible, y asegurarse de que sus compañeros atiendan a los pacientes con tendencia al acoso (por ejemplo, que un colega hombre atienda a un paciente hombre)^(18,35)^.
(2) *Las recomendaciones relacionadas con la institución incluyeron* colgar carteles informativos y motivadores sobre los derechos de los profesionales de la salud, instalar y dar a conocer un sistema de cámaras, contar con un guardia de seguridad especialmente durante el turno nocturno, aumentar el número de personal de seguridad, asegurarse de que el personal de seguridad visite con frecuencia las policlínicas y las habitaciones de los pacientes, organizar capacitación para que el personal esté informado sobre esta situación, garantizar que el personal esté preparado para situaciones inesperadas, planificar cuidadosamente los horarios de trabajo de las empleadas y evitar que trabajen solas por la noche, aumentar el número de personal médico y evitar que el personal femenino se quede a solas con pacientes y familiares, apoyar a la empleada en lugar de al acosador como gerente, introducir reglas estrictas para las visitas de pacientes y garantizar que el agresor enfrente un castigo^(18,35)^.

## DISCUSIÓN

Esta revisión sistemática evaluó 16 artículos (1996–2023) sobre experiencias de acoso sexual entre los profesionales de la salud en Turquía. La mayoría de los estudios se centraron en una región específica (principalmente Ankara), con pocas muestras a nivel nacional, lo que limita la generalizabilidad de los datos de prevalencia. Si bien la mayoría examinó los hospitales del Ministerio de Salud, algunos omitieron detalles institucionales, probablemente para proteger la confidencialidad de los participantes. Esto concuerda con la literatura más amplia, que señala que los estudios a escala nacional a menudo se concentran en ciudades, hospitales o especialidades específicas^(12)^.

La mayoría de los cuestionarios utilizados en los estudios incluyen diferentes herramientas de medición creadas por los autores. Se puede concluir que no existe una herramienta de medición común ampliamente utilizada en la evaluación del acoso sexual entre los profesionales de la salud. Por lo tanto, los métodos de evaluación y las variables de resultado varían de un estudio a otro.

En la mayoría de los estudios analizados, se investigó el acoso sexual junto con otras formas de violencia, y se observó que todos los estudios reportaron tanto altas tasas de acoso sexual como la ausencia de acoso sexual. En la revisión sistemática realizada por Lu et al.^(36)^, que examinó la frecuencia del acoso sexual contra enfermeras en todo el mundo, se constató que la tasa de acoso sexual entre las enfermeras oscilaba entre el 12,6% y el 53,4%. Un estudio realizado entre trabajadores sociales y de la salud en Alemania reveló que el 62,5% de los participantes había sufrido acoso sexual no verbal, el 67,1% había sido objeto de acoso sexual verbal y el 48,9% había sufrido acoso sexual físico perpetrado por pacientes, clientes o residentes en el lugar de trabajo^(37)^. En Italia, se determinó que la prevalencia del acoso sexual era del 2,4% entre las trabajadoras de la salud y del 1,3% entre los trabajadores de la salud^(38)^. En una revisión sistemática realizada para determinar la prevalencia del acoso sexual entre los profesionales de la salud en países de ingresos altos, se determinó que era del 6,0%^(39)^. Las diferencias observadas en los resultados pueden explicarse por variaciones en la percepción del acoso sexual, los contextos culturales y las características estructurales de los entornos en los que se llevaron a cabo los estudios. Debido a la preocupación por el estigma o la exposición pública, es posible que los participantes hayan ocultado información, lo que llevó a los investigadores a analizar todas las formas de violencia en lugar de centrarse únicamente en el acoso sexual. A pesar de las diferentes definiciones, el acoso sexual parece ser frecuente entre los profesionales de la salud en Turquía y requiere mayor atención debido a su impacto negativo en la salud y la calidad de vida.

En los estudios analizados, se observa que la mayoría de las víctimas de acoso sexual son enfermeras, mujeres, jóvenes, solteras y recién contratadas. En una revisión sistemática, se informó que las enfermeras han sufrido acoso sexual en mayor medida que los enfermeros^(40)^; otro estudio indicó que entre el 10,0% y el 87,3% de las enfermeras estuvieron expuestas a acoso sexual en el trabajo^(11)^. En otra revisión sistemática y metaanálisis, se informó que tener menos de 40 años de edad y un estado civil de soltero, separado o viudo se asoció con el acoso sexual^(41)^. En un estudio realizado entre enfermeras en Grecia, se encontró que las enfermeras más jóvenes y solteras eran más propensas a sufrir acoso sexual. Por el contrario, las enfermeras divorciadas con entre 1 y 5 años de experiencia profesional informaron una exposición más frecuente a conductas de coacción sexual^(42)^. En un campo dominado por mujeres, las enfermeras son aún más susceptibles al acoso sexual en el lugar de trabajo, y el acoso sexual contra ellas se está convirtiendo en una amenaza laboral.

Aunque las víctimas de acoso sexual en los estudios analizados en el marco de este trabajo mencionaron a agresores de diferentes grupos, es un hallazgo importante que la mayoría de ellas mencionara a los médicos. En un estudio realizado en Grecia para identificar el acoso sexual entre las enfermeras, se encontró que los médicos varones eran los agresores más frecuentemente reportados^(42)^. En otro estudio realizado en Ghana que examinó el acoso sexual entre enfermeras, se identificó a los médicos hombres como los principales agresores de acoso sexual^(43)^. Según la OMS, los agresores de acoso y violencia pueden ser personas en posiciones de autoridad que gozan de respeto y confianza, como los médicos^(44)^. También cabe destacar que, en otros estudios, la mayoría de los agresores fueron pacientes^(10,12,45)^ y familiares de pacientes^(43,46)^. La mayoría de las muestras de los estudios incluidos en esta revisión consistían en enfermeras, y se puede suponer que estas son más vulnerables debido a las diferencias de poder dentro de las instituciones de salud, donde los médicos ocupan posiciones jerárquicas más altas. Además, las enfermeras pueden estar más expuestas al acoso sexual, ya que tienen contacto y comunicación más frecuentes con otros profesionales de la salud, pacientes y familiares de pacientes, y pasan más horas en el entorno hospitalario.

Los estudios analizados revelaron que los trabajadores de la salud se enfrentaban al acoso sexual con mayor frecuencia en las habitaciones de los pacientes, en áreas no clínicas, en clínicas de hospitalización y durante los turnos nocturnos. Un estudio encontró que la mayor parte del acoso ocurría durante los turnos nocturnos (18:00–07:00)^(47)^, mientras que otro reportó incidentes durante el día (46). De manera similar, un estudio realizado entre trabajadores de la salud en Arabia Saudita informó que los trabajadores del turno nocturno (18:00–07:00) estaban significativamente más expuestos a la violencia sexual^(48)^. En Grecia, los médicos y enfermeras que trabajaban en servicios de emergencia y clínicas ambulatorias tenían 2,696 veces más probabilidades de denunciar acoso sexual en comparación con quienes trabajaban en otros departamentos^(49)^. Trabajar a solas con pacientes aumenta los riesgos para los trabajadores de la salud, particularmente para las enfermeras en las unidades clínicas. Se necesitan sistemas preventivos para garantizar lugares de trabajo más seguros para el personal femenino en las organizaciones de salud.

También cabe destacar que los profesionales de la salud no hacen nada o ignoran el incidente. Los participantes afirmaron que no hicieron ningún intento porque temían las consecuencias, se sentían avergonzados y culpables. Los estudios indican que muchas víctimas, en particular enfermeras y médicos, se abstienen de denunciar el acoso sexual debido al miedo a las consecuencias, la vergüenza, la culpa o la creencia de que no se tomarán medidas^(12,46,50)^. Lu et al.^(36)^ señalaron en su metaanálisis que los profesionales de la salud en Egipto y Turquía se mantenían pasivos ante el acoso sexual, y que las enfermeras, en particular, no denunciaban los incidentes de acoso sexual porque les daba vergüenza. En un estudio cualitativo realizado en Irán que examinó el silencio de las enfermeras ante el acoso sexual, se identificaron cuatro subcategorías: el miedo al estigma social, las barreras organizativas y legales, las barreras relacionadas con la familia y las barreras personales^(51)^. En un estudio, se descubrió que aproximadamente una de cada tres enfermeras no denunciaba el acoso sexual por temor a consecuencias negativas y la creencia de que no se tomarían medidas contra el agresor^(42)^. El acoso sexual es un tema muy delicado en las comunidades conservadoras y puede considerarse una situación vergonzosa de la que hablar. Además, como se menciona en los estudios, el hecho de que los agresores también fueran colegas o personas en puestos de supervisión puede llevar a las víctimas a ocultar el incidente de acoso sexual.

El acoso sexual tiene muchas consecuencias para los profesionales de la salud. En los estudios considerados en el marco de esta revisión sistemática, estas consecuencias se consideraban generalmente físicas y emocionales/psicológicas; sin embargo, también se hizo hincapié en que existían importantes consecuencias organizativas, como la disminución de la productividad, la intención de renunciar al empleo y el cambio de lugar de trabajo. En la literatura, las consecuencias de la violencia sexual también incluyen estrés psicológico, vergüenza, depresión, trastornos del sueño, deterioro del desempeño profesional y relaciones poco saludables e incómodas con los pacientes^(52,53)^. Lu et al.^(36)^ descubrieron que el acoso sexual aumentaba el estrés en las enfermeras, al tiempo que reducía la satisfacción laboral, la calidad del trabajo y la productividad. Chakraborty et al.^(50)^ afirmaron que los incidentes violentos reducían la satisfacción laboral en los profesionales de la salud. Un estudio realizado entre personal paramédico y estudiantes en los Estados Unidos encontró una correlación positiva entre el acoso sexual y el agotamiento profesional^(54)^. Se puede concluir que los hallazgos de este estudio son compatibles con la literatura.

## CONCLUSIONES

En este estudio se presentaron, mediante el método de revisión sistemática, los incidentes de acoso sexual sufridos por los profesionales de la salud en Turquía. Como resultado de la selección, se examinaron 16 estudios que cumplían con los criterios. Se observó que los estudios se realizaron, por lo general, en determinados grupos profesionales, en una sola ciudad y en una sola institución. Todos los estudios fueron estudios descriptivos transversales, que utilizaron, por lo general, cuestionarios desarrollados por los propios investigadores. Por consiguiente, se puede afirmar que no se ha analizado la relación causal entre el incidente de acoso y las reacciones del profesional de la salud que lo sufrió, ni cómo evolucionan los incidentes de acoso sexual a lo largo del tiempo. Además, algunos de los estudios solo analizaron el acoso sexual, mientras que otros lo abordaron desde una perspectiva limitada junto con diferentes tipos de violencia. Aunque, según los estudios analizados, hubo profesionales de la salud que nunca habían sido víctimas de acoso sexual, también hubo estudios que reportaron altas tasas de acoso sexual. Además, un número igual de estudios se centró en todos los profesionales de la salud y en las enfermeras, mientras que pocos se dirigieron exclusivamente a los médicos. No hubo ningún estudio que examinara la incidencia del acoso sexual en un solo grupo de profesionales de la salud, aparte de los médicos y las enfermeras. Los estudios revelaron que los profesionales de la salud experimentaban con frecuencia acoso sexual tanto verbal como físico, a menudo por parte de múltiples agresores, entre ellos colegas, pacientes y sus familiares. Las víctimas, en general, se mantenían pasivas, no denunciaban los incidentes y sufrían consecuencias adversas a nivel físico, psicológico y laboral.

Se requiere una investigación más integral y que utilice múltiples metodologías para desarrollar un modelo holístico que explique las características de los incidentes de acoso sexual que sufren los trabajadores de la salud en Turquía, las respuestas desarrolladas contra el acoso, las estrategias de afrontamiento y los cambios en estas respuestas a lo largo del tiempo. El hecho de que la literatura actual se limite en gran medida a estudios descriptivos dificulta revelar adecuadamente las dimensiones dinámicas y contextuales del fenómeno. Por lo tanto, además de los estudios cuantitativos que permiten comparaciones entre diferentes grupos de profesionales de la salud, tipos de instituciones, ciudades y niveles de servicios de salud, se deben utilizar enfoques de investigación fenomenológicos y otros enfoques cualitativos para garantizar una comprensión más profunda de las experiencias. Dichos estudios contribuirán de manera significativa a comprender la naturaleza multidimensional del acoso sexual y a desarrollar modelos de intervención.

Por otro lado, para prevenir y gestionar eficazmente el acoso sexual contra los trabajadores de la salud, es crucial desarrollar políticas nacionales claras, integrales y vinculantes, establecer sistemas nacionales de recolección de datos que permitan monitorear regularmente la prevalencia del acoso y adoptar enfoques de tolerancia cero respaldados por regulaciones legales. Además, se recomienda implementar programas de capacitación tanto en instituciones educativas como en organizaciones de salud para crear conciencia sobre el acoso sexual entre los profesionales de la salud, los pacientes y sus familiares. A nivel de las instituciones de salud, se sugiere que el tema del acoso sexual se aborde en el marco de los estudios de salud y seguridad ocupacional, que se establezcan mecanismos de denuncia accesibles y seguros, que se brinde un entorno laboral de apoyo a las víctimas, que se implementen procesos legales de manera rápida y eficaz en respuesta a los incidentes, y que se organicen capacitaciones para directivos y empleados a fin de aumentar la concientización institucional. Se considera que estas medidas contribuirán a garantizar la rendición de cuentas y a aumentar el efecto disuasivo respecto al acoso sexual.

## Data Availability

The entire dataset supporting the results of this study was published in the article itself.
